# Projected climate change impacts on habitat suitability of threatened medicinal plants in Himachal Pradesh, Western Himalaya: an ensemble species distribution modelling approach

**DOI:** 10.1038/s41598-026-66433-0

**Published:** 2026-08-17

**Authors:** Simran Tomar, Merja Helena Tölle, K. S. Kanwal, Sunil Puri

**Affiliations:** 1 Institute of Water, Waste and Environmental Engineering, University of Kassel, 34125 Kassel, Germany; 2 School of Biological Sciences, Shoolini University of Biotechnology and Management Sciences, Solan, 173212 India; 3 G. B Pant National Institute of Himalayan Environment, Kosi-Katarmal, Almora, 263643 India

**Keywords:** Threatened species, Species distribution modelling, Ensemble model, Climate change, Climate sciences, Ecology, Ecology, Environmental sciences

## Abstract

Climate change is expected to profoundly influence high-elevation Himalayan ecosystems, where steep environmental gradients and narrow climatic tolerances render alpine plant communities particularly sensitive to environmental changes. Many threatened Himalayan medicinal plants occur near alpine-sub-alpine ecotones associated with upper forest limits, and shifts in their distribution may reflect broader ecosystem responses to climate change. Anticipating such range dynamics is essential for biodiversity conservation and sustainable resource management in climate-sensitive mountain landscapes. We assessed the current and future habitat suitability of three threatened Himalayan medicinal plants, *Aconitum heterophyllum*, *Lilium polyphyllum*, and *Picrorhiza kurroa*, in Himachal Pradesh, Western Himalaya, using an ensemble species distribution modelling framework. Species occurrence records were combined with climatic and topographic predictors to model the current and future habitat suitability. In addition to habitat suitability projections, we integrated model uncertainty, centroid displacement, and climatic niche overlap analyses to assess the robustness and spatial dynamics of future habitat changes. Ensemble models showed strong predictive performance, with the best-performing algorithms (Random Forest and MaxEnt) achieving ROC values of up to 0.90 and TSS values of up to 0.55. Habitat suitability was primarily influenced by precipitation seasonality (BIO15; importance up to ~ 0.45) and the mean diurnal temperature range (BIO2; up to ~ 0.28). Future suitability became increasingly concentrated in high-elevation districts, such as Lahaul and Spiti and Kinnaur, with suitable habitat declining by approximately 33–57% across most future scenarios. *A. heterophyllum* was the only species to show a temporary increase in suitable area (+ 35.7%) under SSP126 in 2050, before declining under later projections. Centroid shifts ranged from 9.4–19.4 km, while Schoener’s D declined from 0.59 to 0.47 across species and scenarios, indicating increasing divergence from current suitability patterns. These findings indicate that climate change may substantially reorganise habitat suitability for threatened Himalayan medicinal plants, highlighting the potential for high-elevation refugia and providing a framework for climate-informed conservation planning in Himachal Pradesh and other Himalayan mountain ecosystems.

**Supplementary Information:**

The online version contains supplementary material available at 10.1038/s41598-026-66433-0.

## Introduction

Climate change is rapidly redefining the structure and function of mountain ecosystems, with profound implications for biodiversity conservation, ecosystem services, and regional livelihoods^1^. The Himalayas, often referred to as the “Third Pole,” is experiencing warming rates that exceed global averages, leading to accelerated cryosphere loss, altered hydrological regimes, and marked shifts in vegetation composition^2–5^. These changes have important conservation implications, as climate-induced redistribution of alpine flora threatens not only endemic biodiversity but also culturally and economically important medicinal plant resources that underpin traditional healthcare systems and contribute substantially to rural livelihoods^6^. Therefore, effective conservation and adaptation planning in such climatically sensitive regions therefore requires spatially explicit, forward-looking assessments that can reliably inform management decisions under uncertainty.

Recent ecological studies have indicated that climate warming is already altering the functioning of Himalayan alpine plant communities through multiple pathways beyond distributional change. Earlier snowmelt and changing temperature regimes have been shown to advance flowering phenology and alter reproductive timing in Himalayan plant species, including climatically sensitive taxa from the Indian Himalayas^7,8^. Changes in snowmelt timing and temperature regimes have also been linked to shifts in species interactions and ecosystem functioning, highlighting the sensitivity of high-elevation Himalayan vegetation to ongoing climatic changes^9,10^. Experimental and observational studies have further demonstrated that warming can drive changes in plant functional traits, community composition, and vegetation structure, potentially favouring species with broader climatic tolerances while increasing the vulnerability of specialized alpine taxa^11,12^. Together, these studies demonstrate that climate warming is already influencing the ecological processes underpinning species persistence and habitat occupancy in Himalayan mountain ecosystems. Therefore, assessing projected changes in climatic suitability therefore complements existing ecological evidence and provides an important basis for anticipating future conservation challenges for threatened medicinal plants.

High-elevation medicinal plant species in Himachal Pradesh are particularly vulnerable to climate change because many occupy climatically restricted alpine and sub-alpine habitats within the state, exhibit narrow environmental tolerances, and depend strongly on temperature and precipitation regimes that are undergoing rapid change in Himalayan mountain ecosystems^13,14^. Species such as *Aconitum heterophyllum, Lilium polyphyllum*, and *Picrorhiza kurroa* are valued for their pharmacological properties and are experiencing population declines driven by overharvesting, habitat degradation, and limited natural regeneration^15–18^. Climate change is expected to exacerbate these pressures by reshaping the climatic suitability and geographic distribution of their habitats^19^. Understanding how these species may respond to future climatic change is therefore essential for anticipating conservation risks and designing proactive management strategies.

Species distribution models (SDMs) are widely used to assess current habitat suitability and to project potential shifts in species distributions under future climate scenarios. Ensemble modelling approaches combine predictions from multiple algorithms to reduce model-specific bias and generally provide more robust estimates of habitat suitability than individual algorithms^20–23^. However, reliable conservation planning also requires an assessment of prediction uncertainty and spatial redistribution under future climate conditions. Integrating ensemble suitability projections with complementary spatial metrics, such as model uncertainty, centroid displacement, and niche overlap, provides additional insight into the confidence and ecological implications of the projected distributional changes. Despite their potential value, such integrated assessments remain limited for threatened medicinal plants in the Western Himalayas.

In this study, we applied an ensemble species distribution modelling framework to evaluate the current and future habitat suitability of *A. heterophyllum, L. polyphyllum*, and *P. kurroa* across Himachal Pradesh in the Western Himalaya. Using six modelling algorithms implemented in BIOMOD2, representing both statistical and machine-learning approaches, we generated ensemble projections under three Shared Socioeconomic Pathways (SSP126, SSP245, and SSP585) for mid-century (2050) and late-century (2070) climate conditions. In addition to ensemble habitat suitability projections, we evaluated model uncertainty and complementary spatial metrics, including centroid displacement and Schoener’s D niche overlap, to better characterise projected distributional changes and identify areas of potential climatic persistence. However, the projections represent climatic suitability only and do not explicitly account for biotic interactions, dispersal limitations, disease dynamics, species-area constraints, or the formation of novel ecological communities under climate change. Consequently, projected suitability should be interpreted as an indicator of potential climatic opportunity rather than a realised future species distribution.

Based on observed warming trends, changing snow dynamics, and the ecological sensitivity of alpine medicinal plants, we hypothesised that (i) projected climatically suitable habitats for all three species would contract and become increasingly concentrated in higher-elevation areas of Himachal Pradesh; (ii) habitat contraction and spatial redistribution would be greatest under higher-emission scenarios; and (iii) future suitability patterns would increasingly diverge from current conditions, as reflected by centroid displacement and declining niche overlap. Accordingly, the objectives of this study were to: (i) evaluate the current climatic suitability of *A. heterophyllum*, *L. polyphyllum*, and *P. kurroa* in Himachal Pradesh using an ensemble species distribution modelling framework;

(ii) project changes in climatic suitability under SSP126, SSP245, and SSP585 climate scenarios for 2050 and 2070;

(iii) identify areas of habitat gain, loss, and persistence under future climate conditions;

(iv) quantify model uncertainty associated with ensemble projections; and.

(v) assess the spatial redistribution of climatically suitable habitats using centroid displacement and Schoener’s D niche overlap metrics.

## Materials and methods

### Study area

**Fig. 1:**
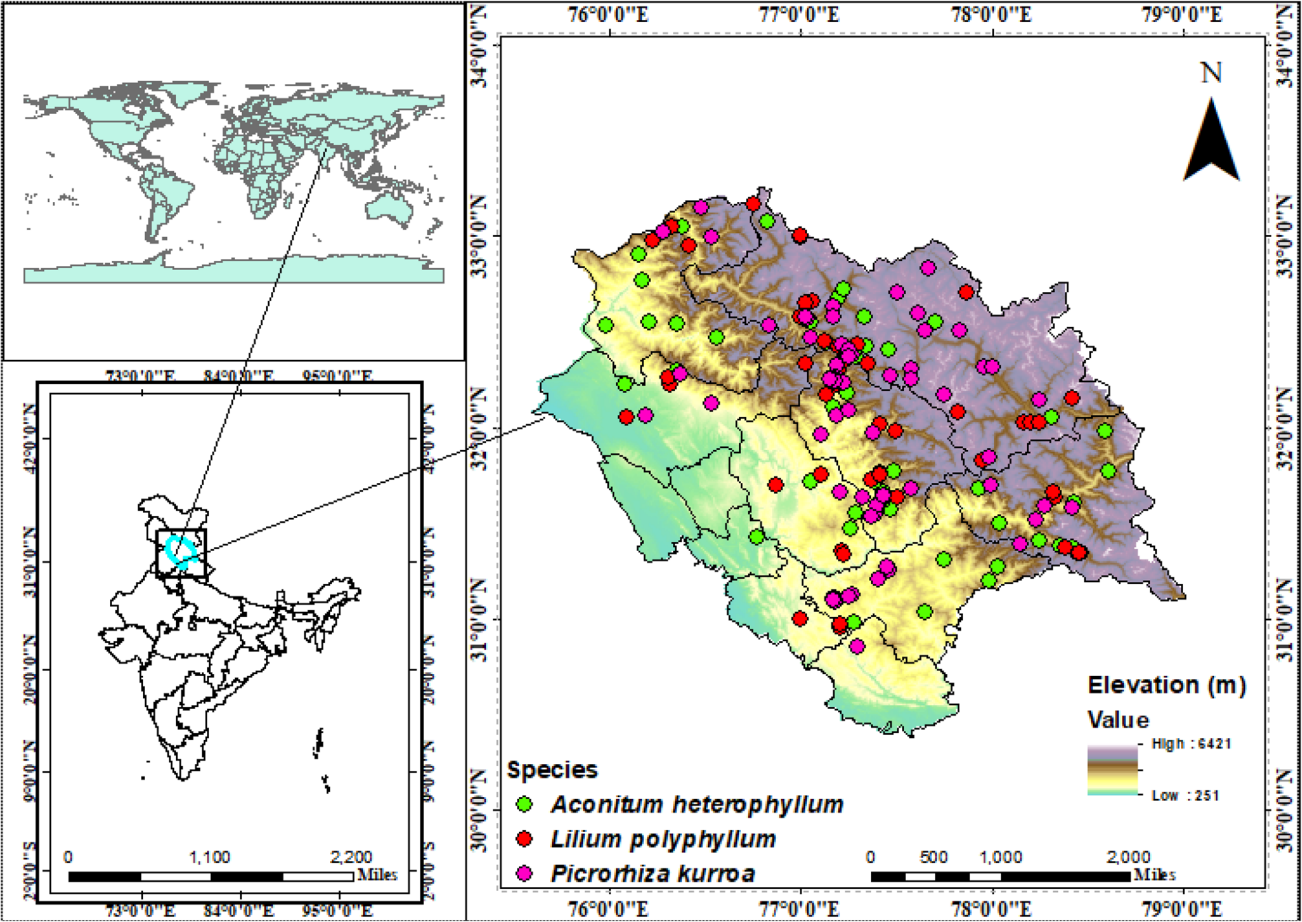
Study area map of Kullu district showing the sampling locations of *A. heterophyllum*, *L. polyphyllum* and *P. kurroa*.

This study was conducted in the sub-alpine and alpine regions of Himachal Pradesh, India (Fig. 1). Himachal Pradesh was selected as the modelling extent because it represents one of the most ecologically heterogeneous sectors of the Western Indian Himalaya and is recognised as an important centre of Himalayan plant diversity. The state encompasses strong elevation gradients and climatic variability that support ecosystems ranging from subtropical forests to alpine meadows and cold desert environments. This environmental heterogeneity supports exceptionally rich floristic diversity, with approximately 3,400 vascular plant species, including more than 1,000 medicinal and aromatic plant species, many of which are endemic, near-endemic, or threatened^24,25^. Recent studies have highlighted that Himalayan mountain ecosystems, including those of Himachal Pradesh, are highly sensitive to climate warming, exhibiting elevational shifts in vegetation, altered snow dynamics, and increasing pressure on high-altitude medicinal plants^9,10,26,27^. These characteristics make Himachal Pradesh an ideal region for assessing climate-driven distributional changes using species-distribution models. All three focal species (*A. heterophyllum*, *L. polyphyllum*, and *P. kurroa*) occur naturally within Himachal Pradesh across broad elevation gradients, providing a robust regional framework for evaluating current and future habitat suitability. The three focal species are primarily associated with sub-alpine and alpine habitats, typically occurring between approximately 2,500 and 5,000 m above sea level. in Himachal Pradesh, where climatic conditions strongly influence their distribution and persistence. Himachal Pradesh also represents a well-defined administrative unit where biodiversity conservation, forest management, and medicinal plant conservation programs are actively implemented, making the modelling outputs directly relevant for regional conservation planning. Accordingly, the present study was designed as a regional assessment confined to Himachal Pradesh, and the resulting projections should be interpreted within this geographical extent rather than extrapolated to the entire Western Himalaya. The study area encompasses sub-alpine forests, alpine meadows, alpine scrub, and cold desert ecosystems that provide important habitats for high-elevation medicinal plant species^28^. The climate in Himachal Pradesh is influenced by Western disturbances, convective storms and air currents from Bay of Bengal^29^. The mean annual air temperatures across Himachal Pradesh range from approximately 5 °C to 20 °C^30^. The region exhibits pronounced local and regional topographic variations resulting from multiple past glaciation events^31^. The study area forms part of the Himalayan biodiversity Hotspot, one of the world’s recognized biodiversity hotspots, and is considered highly sensitive and vulnerable to ongoing climate change^32–34^. The steep climatic gradients, high topographic complexity, and documented sensitivity of Himalayan ecosystems to recent warming provide an ideal natural setting for investigating climate-driven changes in habitat suitability and identifying potential conservation priorities for the threatened medicinal plants.

### Study species

This study focused on three threatened Himalayan medicinal plant species: *A. heterophyllum*, *L. polyphyllum*, and *P. kurroa*. These species were selected because they are ecologically associated with the alpine and sub-alpine habitats of Himachal Pradesh, possess high medicinal value, and have experienced population declines due to overharvesting, habitat degradation, restricted regeneration, and climate-related pressures. All three species are recognised as conservation priorities in Himachal Pradesh and are considered highly vulnerable to environmental changes because of their specialised habitat requirements and restricted elevational distributions. A summary of their ecological characteristics, conservation status, and major threats is presented in Table 1**.**

**Table 1:** Ecological and conservation characteristics of the study species.

Species	Family	Medicinal importance	Habitat & elevational range	Regional status	Global status*	Global distribution	Major threats
*A. heterophyllum* Wall. ex Royle	Ranunculaceae	Widely used in traditional medicine for fever, digestive disorders and respiratory ailments	Alpine and sub-alpine meadows, rocky slopes; ~ 2,500–4,500 m	CR	EN; A2cd	India, Nepal, Pakistan, Tibet	Overharvesting, habitat degradation, low regeneration, climate change
*L. polyphyllum* D. Don	Liliaceae	Important medicinal and ornamental species; bulbs used in traditional medicine	Open alpine meadows and forest clearings; ~ 2,400–4,000 m	CR	CR; A2cd	India, Nepal, Pakistan, Afghanistan, Myanmar	Habitat loss, collection pressure, poor natural regeneration, climate change
*P. kurroa* Royle ex Benth	Plantaginaceae	High-value medicinal herb used for liver disorders and immune-related treatments	Alpine slopes, moist rocky habitats; ~ 3,000–5,000 m	CR	EN; A2cd	India, Pakistan, Nepal, Bhutan, Myanmar	Commercial extraction, habitat degradation, climate warming, restricted habitat availability

(Abbreviations**:** CR = Critically Endangered; EN = Endangered. Conservation status follows the IUCN Red List assessments (*Aconitum heterophyllum* – Endangered, 2014; *Lilium polyphyllum* – Critically Endangered, 2015; *Picrorhiza kurroa* – Endangered, 2021) and the Himachal Pradesh Conservation Assessment and Management Prioritisation (CAMP) assessment^78^. Information on medicinal uses, habitat preferences, elevational ranges, and threats was compiled from published literature source:^16,78,79,80^; . Abbreviations: CR = Critically Endangered; EN = Endangered.

### Species occurrence data and environmental predictors

Field-based occurrence records were compiled from non-destructive botanical surveys conducted between 2018 and 2024 across the sub-alpine and alpine regions of Himachal Pradesh. Surveys were conducted during the growing season when the focal species could be reliably identified using diagnostic vegetative and reproductive characteristics. Geographic coordinates were recorded in the field using handheld GPS devices in the WGS84 geographic coordinate reference system and subsequently checked for positional consistency before being included in the modelling dataset. As the objective of the fieldwork was to document species occurrences rather than estimate population abundance, observations were incorporated as georeferenced presence records for species distribution modelling. Surveys focused on the known and potential habitats of the target species, including alpine meadows, rocky slopes, forest clearings, and moist alpine habitats. No plant material was collected during field surveys because all focal species are of conservation concern within Himachal Pradesh, and the study relied exclusively on non-destructive observations. Species identification was based on diagnostic morphological characteristics described in regional floras and taxonomic literature and was verified through comparison with authenticated herbarium specimens and type-image resources available through the G.B. Pant National Institute of Himalayan Environment, Almora, Uttarakhand, India. Field photographs were examined to confirm key distinguishing characteristics and minimise potential misidentification with morphologically similar taxa in the region. Representative field photographs of the three focal species are shown in Fig. S1; Supplementary File 1). Species presence records for *A. heterophyllum, L. polyphyllum*, and *P. kurroa* were compiled from several sources. Occurrence records were compiled from field surveys (2018–2024), georeferenced herbarium specimens, published literature, and the Global Biodiversity Information Facility (GBIF; http://www.gbif.org/) (Table S1; Supplementary File 1). Species occurrence records from all sources were screened for data quality prior to the modelling. Duplicate records, records lacking geographic coordinates, records falling outside the known elevational or geographic range of the species, and records with obvious geographic errors (e.g. coordinates outside Himachal Pradesh) were removed prior to the modelling. To reduce spatial clustering and sampling bias arising from uneven sampling effort among data sources, duplicate records and multiple occurrences located within the same 1-km raster cell were removed prior to modelling, retaining only a single occurrence per grid-cell. Because all environmental predictors were analysed at a spatial resolution of approximately 1 km, this procedure reduced the influence of redundant records originating from multiple data sources. Following data cleaning and duplicate removal, the final dataset consisted of 73 occurrence records for *A. heterophyllum*, 73 for *L. polyphyllum*, and 77 for *P. kurroa*. For herbarium and literature records lacking geographic coordinates, the locations were georeferenced using locality descriptions, gazetteers, and digital mapping resources. Records for which the geographic coordinates could not be assigned with sufficient confidence were excluded. Field surveys were designed to document species occurrences rather than systematically record their absence across a standardised sampling framework. Consequently, reliable absence data were unavailable, necessitating the use of pseudo-absence generation for modelling algorithms that require both presence and absence information. Because reliable absence records were unavailable for the study species, pseudo-absence data were generated using the disk strategy implemented in the BIOMOD2. For each species, the number of pseudo-absence points was set equal to the number of presence records. Pseudo-absence locations were selected between a minimum distance of 5 km and a maximum distance of 50 km from known occurrence records, thereby avoiding pseudo-absences immediately adjacent to presence while restricting selection to environmentally accessible areas. The 5 km minimum distance reduced the likelihood of assigning pseudo-absences within potentially suitable habitats immediately surrounding known occurrences, whereas the 50 km maximum distance restricted pseudo-absence selection to regions considered environmentally accessible to the species. Two independent pseudo-absence datasets were generated for each species to account for the uncertainty associated with random pseudo-absence selection. Although a larger number of pseudo-absence replicates can be used, previous studies have shown that a limited number of well-distributed pseudo-absence datasets is generally sufficient when combined with repeated model evaluation and ensemble forecasting, as the influence of any individual pseudo-absence selection is reduced through model averaging^35–37^. The two pseudo-absence replicates were subsequently incorporated into the ensemble modelling workflow, together with repeated model runs and performance-based ensemble construction, to produce stable predictions across algorithms and climate scenarios.

A total of 24 environmental variables were initially assembled for the species distribution modelling. These included 19 bioclimatic variables (BIO1–BIO19) (https://www.worldclim.org/data/bioclim.html) and elevation (https://www.worldclim.org/data/worldclim21.html) from the WorldClim database version 2.1. Land cover was downloaded from Shuttle Radar Topographic Data (SRTM) at a spatial resolution of 30 arc-seconds. The slope and aspect were derived from the elevation data. Soil cover data was obtained from the Harmonised World Soil Database (HWSD) of the Food and Agricultural Organization of the United Nations (FAO) soil portal (https://www.fao.org/soils-portal/data-hub/soil-maps-and-databases/harmonized-world-soil-database-v12/en/). All layers were resampled to a common 1 km resolution, stacked, projected, and masked to the extent of Himachal Pradesh using the Raster package. To reduce multicollinearity among environmental predictors, the initial set of 24 environmental variables (19 bioclimatic variables, elevation, slope, aspect, land cover, and soil type) was subjected to Spearman rank correlation analysis. Variables exhibiting pairwise correlations greater than |0.70| were considered highly correlated and were removed. The remaining variables were subsequently evaluated using Variance Inflation Factor (VIF) analysis, and only variables with VIF values < 10 were retained^38^. This procedure resulted in a final set of eight predictor variables (BIO2, BIO3, BIO14, BIO15, slope, aspect, land cover, and soil type). The correlation results, variables removed during screening, and VIF values for the retained predictors are provided in Table S2 (Supplementary File 1). The retained variables were selected based on their ecological relevance to the distribution of alpine medicinal plants in the Himalayas. The retained variables were selected because they represent the major climatic, topographic, and habitat gradients that influence alpine plant distribution. BIO2 and BIO3 describe temperature variability and thermal stability, and BIO14 and BIO15 characterise moisture availability and precipitation seasonality, whereas slope and aspect influence local microclimatic conditions through their effects on solar radiation and snow dynamics. Land cover and soil variables represent habitat and substrate characteristics that affect species establishment and persistence.

### Species distribution modelling

Species distributions were modelled using an ensemble species distribution modelling framework implemented in the BIOMOD2 package^37^, which integrates multiple modelling algorithms and evaluation procedures. Six modelling algorithms implemented in BIOMOD2 (GLM, GAM, RF, MARS, CTA, and MAXENT) were used to generate ensemble projections under three Shared Socioeconomic Pathways (SSP126, SSP245, and SSP585) for mid-century (2050) and late-century (2070) climate conditions. These algorithms were selected because they represent a diverse range of modelling approaches commonly used in species distribution modelling, including regression-based methods (GLM, GAM), tree-based approaches (CTA, RF), flexible non-linear modelling techniques (MARS), and presence-background machine-learning models (MAXENT). Combining algorithms with different underlying assumptions reduces dependence on any single modelling framework and improves the robustness of the ensemble predictions. Previous studies have shown that ensemble forecasts derived from multiple algorithm classes generally provide more reliable estimates of species–environment relationships and future habitat suitability than individual models^21,36^. Several algorithms available in BIOMOD2 were not included because they are less frequently used in contemporary ecological applications, may exhibit unstable performance with relatively small occurrence datasets, or provide information that is largely redundant with the selected methods. Model parameterisation generally followed the default settings implemented in BIOMOD2, with the exception of GAM models, for which a smoothing parameter (k = 3) was specified. No additional algorithm-specific tuning procedures were used. Model calibration used 75% of the data, with 5-fold cross-validation repeated across five iterations. Model performance was evaluated using the Area Under the Receiver Operating Characteristic Curve (ROC) and the True Skill Statistic (TSS). Both metrics were calculated during the model calibration and cross-validation. Ensemble model construction was based on ROC values, and only models with ROC ≥ 0.7 were retained for ensemble development^39,40^. TSS values were used as a complementary measure of predictive performance and for comparison among the modelling algorithms but were not used as a formal criterion for ensemble selection. Because the modelling workflow incorporated pseudo-absence data rather than presence-only models, the ROC and TSS were considered appropriate measures of discriminatory performance.

To evaluate the contribution of each predictor variable, a three-run permutation analysis was conducted^36,37^. This provides a robust estimate of the variable importance across the modelling runs. The scores were extracted and averaged across the models to identify the most influential predictors. These scores are algorithm-independent and are scaled from 0 to 1, with higher values indicating stronger predictive power.

To examine species–environment relationships, partial dependence plots (PDPs) were generated for the three most influential predictors identified using permutation-based variable importance analysis. PDPs were derived from the highest-performing model within each algorithm based on the ROC scores and were used to visualize the marginal effects of environmental predictors on habitat suitability^36,37,41,42^.

Ensemble projections were generated using the Committee Averaging (EMca) and Weighted Mean (EMwmean) approaches implemented in the BIOMOD2. Ensemble suitability outputs were produced as continuous habitat suitability surfaces ranging from 0 to 1. For visualisation and spatial analysis, suitability values were classified into four ordinal categories (unsuitable, low, medium, and high) using quantile-based thresholds. Reclassified maps were exported as GeoTIFF and PNG files for further analysis and visualisation^36,43,44^.

To quantify the spatial distribution of habitat suitability across Himachal Pradesh, district boundaries were overlaid on the reclassified suitability maps, and the area (km^2^) occupied by each suitability class was calculated using the exactextractr package in R^45^. These summaries were used to compare the projected habitat suitability among districts and identify regions of potential conservation importance.

### Future projections under climate change

Future climatic suitability was projected for* A. heterophyllum*, *L. polyphyllum,* and *P. kurroa* using downscaled CMIP6 climate data from the HadGEM global circulation model (GCM) obtained from the WorldClim v2.1 database at an approximate 1-km spatial resolution. Projections were generated for three Shared Socioeconomic Pathways (SSP126, SSP245, and SSP585), representing low-, intermediate-, and high-emission futures for mid-century (2050) and late-century (2070) periods.

HadGEM3-GC31-LL was selected because it has been widely used in climate-impact and species distribution modelling studies and is available as a high-resolution downscaled CMIP6 dataset through the WorldClim v2.1 database^46,47^. Previous studies in the Himalayan region have also demonstrated its suitability for modelling plant distributions across complex mountain climatic gradients^48^.

Future projections were generated using climatic variables, while land cover and soil layers were assumed to remain constant over time because reliable future projections for these variables were unavailable at comparable spatial resolutions. Consequently, projected suitability patterns should be interpreted as responses to climatic change under current land cover and soil conditions. In addition, the 1-km spatial resolution used in this study may not fully capture the fine-scale topographic and microclimatic heterogeneity characteristic of Himalayan alpine environments.

All analyses were conducted in R version 4.2.2, R Core Team, 2022, using the BIOmod2^37^, raster^49^, terra^50^, rgdal^51^, sp^52^, dplyr^53^, ggplot2^54^, and pdp^55^ packages. MaxEnt models were implemented using MaxEnt version 3.4.4^56^ through the maxent.jar application under Java 1.8. All spatial analyses were performed using the WGS84 coordinate reference system.

### Mean ensemble suitability and model uncertainty

Ensemble suitability and uncertainty maps were generated to summarise habitat suitability patterns and quantify algorithmic uncertainty. Ensemble suitability projections produced by BIOMOD2 represent a performance-weighted consensus of the individual modelling algorithms (GLM, GAM, RF, MARS, CTA, and MAXENT), with model contributions weighted according to the evaluation performance.

Mean suitability maps were generated by calculating the arithmetic mean of the suitability values predicted by all individual algorithms for each raster cell. These maps provide a descriptive representation of the average habitat suitability across the modelling approaches.

Model uncertainty was quantified as the standard deviation (SD) of the suitability predictions generated by the individual algorithms for each raster cell^43,44^. Low SD values indicate strong agreement among the algorithms and, therefore, greater confidence in the predicted suitability, whereas high SD values indicate greater disagreement among the algorithms and increased prediction uncertainty. Uncertainty maps were used to identify regions in which the projected habitat suitability was associated with higher levels of algorithmic uncertainty.

### Future range shift and centroid analysis

Centroid analyses were conducted using ensemble suitability projections generated by BIOMOD2 to quantify potential spatial shifts in habitat suitability under climate change. For each species, a suitability-weighted geographic centroid was calculated from the current and future ensemble projections under SSP126, SSP245, and SSP585 for 2050 and 2070^57,58^. Therefore, the centroid represents the geographic centre of the predicted habitat suitability rather than the geometric centre of the species range. The range-shift metrics included: (i) centroid displacement (km), calculated as the straight-line distance between current and future centroids; (ii) bearing (degrees), representing the direction of centroid movement relative to geographic north; and (iii) Schoener’s D, which quantifies niche overlap between current and future suitability projections, ranging from 0 (no overlap) to 1 (complete overlap)^59^.

## Results

### Niche model performance

The six modelling algorithms showed generally good predictive performance across all three species, with ROC values mostly exceeding 0.70 and TSS values indicating moderate discriminatory ability (Fig. S2; Supplementary File 1). Across species, Random Forest consistently achieved the highest median ROC and TSS values and showed comparatively stable performance across model runs. MaxEnt, GAM, GLM, and MARS showed intermediate predictive accuracy, whereas CTA generally produced the lowest evaluation scores.

For *A. heterophyllum* and *L. polyphyllum*, RF achieved the highest predictive performance, with median ROC values approaching 0.85–0.90 and TSS values exceeding 0.45. Similar patterns were observed for *P. kurroa*, although overall evaluation scores were slightly lower than those obtained for the other two species. Despite differences among algorithms, most models yielded ROC values above 0.70, indicating acceptable predictive performance.

Based on these evaluation metrics, RF and MaxEnt were among the best-performing algorithms and contributed strongly to the final ensemble projections.

### Environmental variables and habitat suitability prediction

Environmental predictor importance varied among species, although some common patterns were observed (Fig. S3; Supplementary File 1). For *A. heterophyllum*, precipitation seasonality (BIO15) was the most influential predictor (~ 0.45), followed by mean diurnal range (BIO2; ~ 0.22) and soil (~ 0.12). All remaining variables contributed less than 0.10.

For *L. polyphyllum*, precipitation seasonality (BIO15; ~ 0.34) and mean diurnal range (BIO2; ~ 0.28) were also the most important predictors, followed by aspect and land cover (~ 0.10–0.12). Soil and isothermality (BIO3) showed intermediate importance, while slope and precipitation of the driest month (BIO14) contributed less than 0.06.

For *P. kurroa*, precipitation seasonality (BIO15; ~ 0.35) and mean diurnal range (BIO2; ~ 0.28) were the dominant predictors, followed by soil (~ 0.18). Land cover and isothermality (BIO3) showed moderate importance, whereas slope, BIO14 and aspect each contributed less than 0.08.

Across all species, precipitation seasonality (BIO15) and mean diurnal range (BIO2) consistently ranked among the most influential predictors, while topographic variables generally showed lower contributions.

### Response of habitat suitability to key environmental predictors based on partial dependence plots

Partial dependence plots (PDPs) derived from the Random Forest models illustrated species-specific responses to the most influential environmental predictors.

For *A. heterophyllum*, habitat suitability increased with precipitation seasonality (BIO15) and mean diurnal range (BIO2), while isothermality (BIO3) exhibited a unimodal response with peak suitability at intermediate values.

For *L. polyphyllum*, habitat suitability declined with increasing precipitation seasonality (BIO15) and isothermality (BIO3), whereas suitability increased gradually with precipitation of the driest month (BIO14).

For *P. kurroa*, habitat suitability increased with precipitation seasonality (BIO15) and mean diurnal range (BIO2). Aspect also influenced suitability, with higher predicted suitability occurring at intermediate to higher aspect values.

Overall, precipitation seasonality (BIO15) was a key predictor for all three species; however, the direction of the response differed among species, particularly for *L. polyphyllum,* which showed decreasing suitability with increasing seasonality. The full set of partial dependence plots is provided in Fig. S4; Supplementary File 1.

### Current distribution of *A. Heterophyllum, L. polyphyllum* and *P. kurroa*

**Fig. 2:**
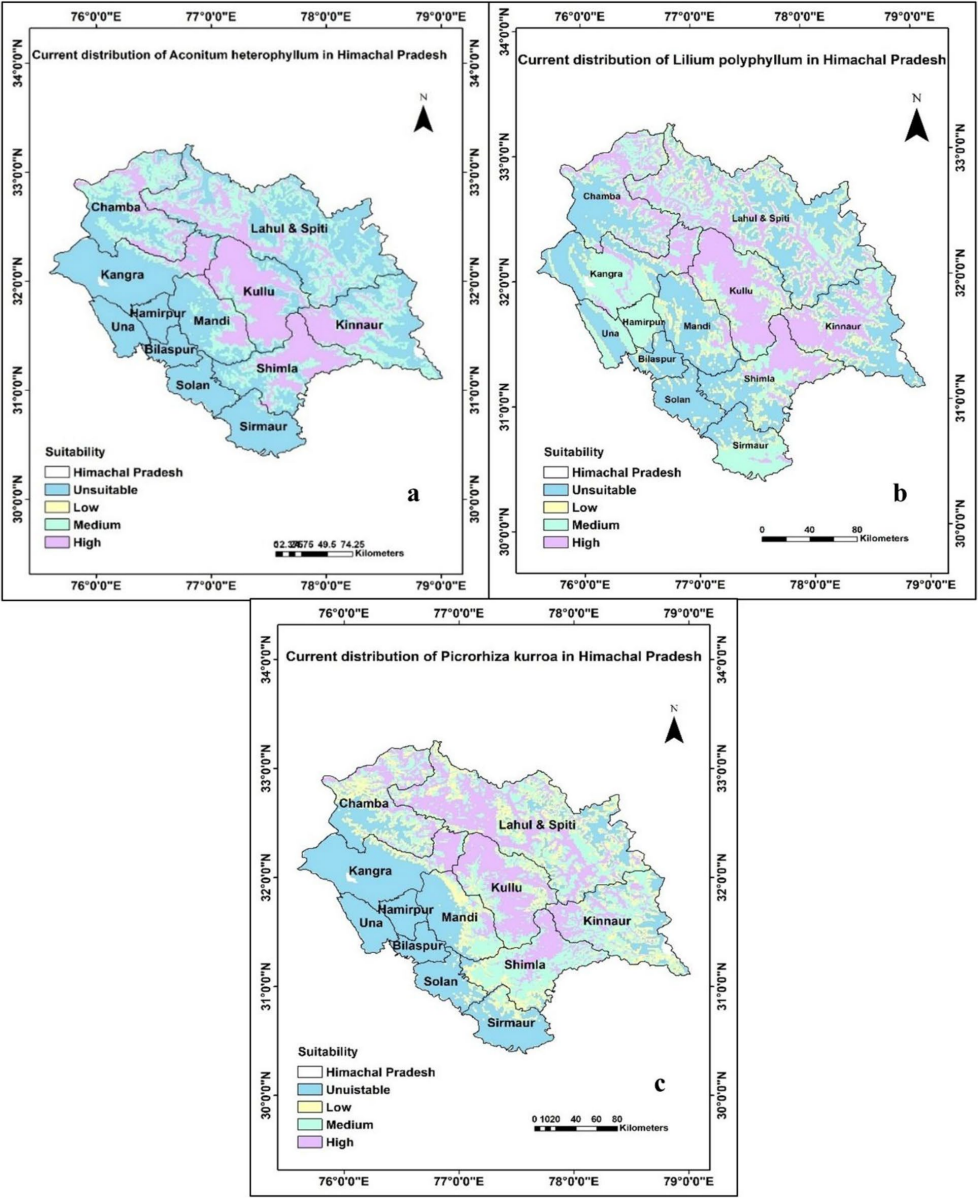
Current habitat suitability of (**a**) *A. heterophyllum*, (**b**) *L. polyphyllum*, and (**c**) *P. kurroa* in Himachal Pradesh.

The ensemble habitat suitability maps revealed distinct but partially overlapping distribution patterns for the three medicinal plant species across Himachal Pradesh (Fig. 2) (Table S3; Supplementary File 1). Suitable habitats were primarily concentrated in the central and high-elevation regions of the state, whereas low-elevation areas showed limited suitability.

*A. heterophyllum* exhibited the most restricted distribution, with 27,406.67 km^2^ classified as suitable habitat and 26,809.52 km^2^ as unsuitable. Suitable areas were concentrated mainly in high-altitude regions, indicating a relatively narrow spatial distribution.

*L. polyphyllum* showed the broadest distribution among the three species, with 35,215.18 km^2^ of suitable habitat and 20,342.06 km^2^ of unsuitable area. Suitable habitats extended across a broader elevational range than those of *A. heterophyllum.*

*P. kurroa* occupied 36,298.68 km^2^ of suitable habitat and 18,631.97 km^2^ of unsuitable area. Although the total suitable area was comparable to that of *L. polyphyllum,* suitable habitats were largely concentrated within alpine and high-elevation regions.

Overall, suitability for all three species was concentrated in the higher-elevation parts of Himachal Pradesh, with substantial overlap in major habitat hotspots. In contrast, low-elevation regions consistently showed low habitat suitability. District-level suitability estimates are provided in the Supplementary File 1.

### Future habitat projections under climate change

**Fig. 3:**
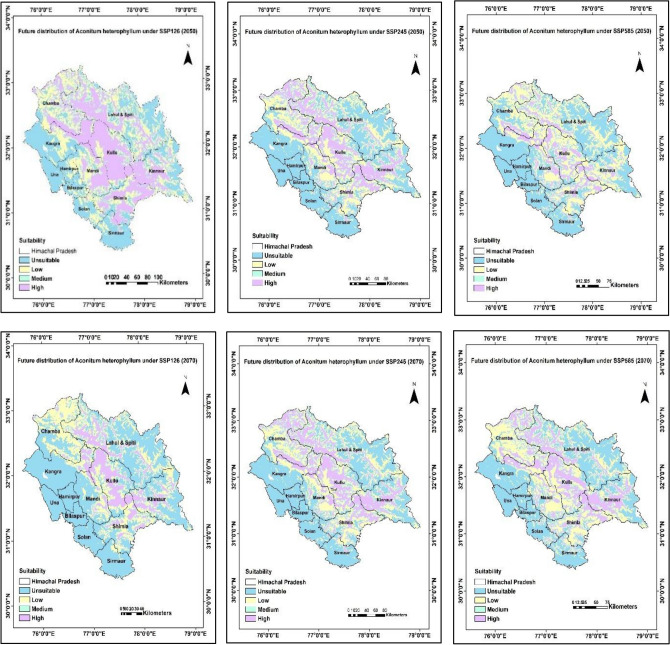
Future habitat projections of *A. heterophyllum.*

**Fig. 4:**
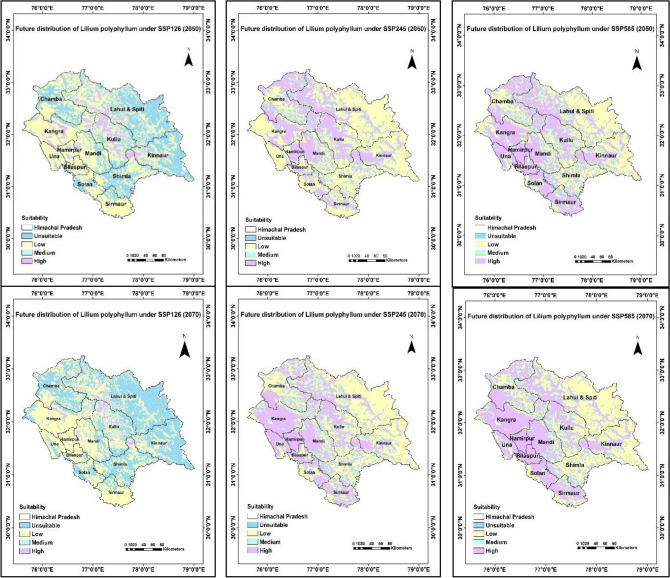
Future habitat projections of *L. polyphyllum*.

**Fig. 5:**
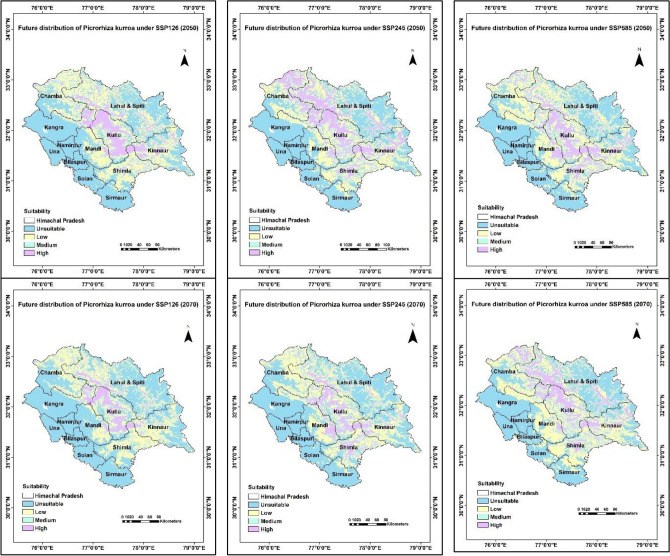
Future habitat projections of *P. kurroa.*

Future projections revealed consistent climate-driven shifts in habitat suitability across all three species, although the magnitude of change differed among taxa (Fig. 3, 4, 5) (Table S4; Supplementary File 2). Under SSP126, suitable habitats remained relatively stable through 2050 and 2070, with persistence of medium- and high-suitability areas in the higher-elevation regions of Himachal Pradesh. Habitat contraction became more evident under SSP245 and intensified under SSP585, particularly by 2070.

Among the three species, *L. polyphyllum* exhibited the greatest reduction and fragmentation of suitable habitat under future climates, whereas *P. kurroa* retained the largest proportion of suitable area across scenarios. *A. heterophyllum* showed an intermediate response, with progressive contraction of suitable habitat under increasing warming. Across all species, future suitability became increasingly concentrated in higher-elevation areas, while lower-elevation regions showed declining suitability.

The strongest changes occurred under SSP585 by 2070, where suitable habitats were substantially reduced and spatially fragmented. In contrast, SSP126 projections indicated comparatively limited changes, suggesting that lower-emission pathways may support greater persistence of suitable habitat. Overall, the results indicate increasing habitat contraction and redistribution with increasing warming intensity, with species-specific differences in the magnitude of projected change.

### Mean ensemble suitability and model uncertainty under future climate scenarios

Ensemble projections, mean suitability maps, and uncertainty maps showed consistent spatial patterns across all three species under future climate scenarios (Fig. S6(a-c); Supplementary File 2). Ensemble and mean projections generally indicated persistence of suitable habitat in higher-elevation regions under SSP126, whereas SSP245 and SSP585 projected progressive reductions and spatial shifts in suitability, particularly by 2070. Uncertainty maps represented variation among SDM algorithms included in the ensemble framework and therefore reflected algorithmic uncertainty rather than climate-model uncertainty, as projections were based on a single GCM.

For *A. heterophyllum*, suitable habitat remained relatively stable under SSP126 but progressively contracted under SSP245 and SSP585. By 2070, SSP585 projections showed suitability largely restricted to high-elevation areas. Algorithmic uncertainty was generally low in core suitable habitats and higher in transitional zones, with the greatest uncertainty observed under SSP585.

*L. polyphyllum* exhibited a similar pattern, with broad habitat retention under SSP126 and increasing contraction under SSP245 and SSP585. Suitable areas became progressively restricted to higher elevations by 2070, particularly under SSP585. Uncertainty remained low in areas consistently predicted as suitable but increased in regions undergoing suitability transitions.

For *P. kurroa*, suitable habitat remained comparatively stable under SSP126 but declined under SSP245 and SSP585. By 2070, high-emission scenarios projected suitability largely confined to the highest elevation areas. Algorithmic uncertainty was lowest in persistent alpine habitats and highest along lower-elevation distribution margins.

Across all species, uncertainty increased with emission intensity and was highest under SSP585 by 2070. Areas of persistent suitability showed strong agreement among modelling algorithms, whereas transition zones exhibited greater disagreement. These results indicate increasing habitat contraction and redistribution under future climate change, accompanied by increasing algorithmic uncertainty under more severe warming scenarios.

### Centroid shifts, bearing changes, and niche overlap under future climate scenarios

Projected climate change resulted in measurable shifts in habitat centroids and reductions in niche overlap for all three species (Supplementary Table S5). Centroid displacement was smallest for *A. heterophyllum* (approximately 9–12 km), intermediate for *P. kurroa* (13–16 km), and greatest for *L. polyphyllum* (14–19 km) across scenarios and time periods.

All species exhibited broadly south-westward centroid shifts, with bearings ranging from approximately -97° to -149°. The direction of movement remained relatively consistent across SSP126, SSP245, and SSP585, although displacement distances generally increased under higher-emission scenarios and later time periods.

Niche overlap between current and future suitability declined across all species and scenarios. Schoener’s D decreased from approximately 0.56 to 0.49 for *A. heterophyllum*, from 0.59 to 0.53 for *L. polyphyllum*, and from 0.50 to 0.47 for *P. kurroa*. The largest reductions were observed under SSP585 by 2070.

Overall, the results indicate increasing divergence between current and future suitable habitats, reflected by both centroid displacement and declining niche overlap under future climate scenarios.

## Discussion

The ensemble modelling framework demonstrated strong predictive performance across algorithms, providing confidence in the projected patterns of current and future habitat suitability for *A. heterophyllum*, *L. polyphyllum*, and *P. kurroa*. High ROC and TSS values obtained for the ensemble outputs support the use of multi-algorithm approaches for reducing individual model bias and improving predictive reliability in mountain ecosystems^44,44,60^. The close agreement between ensemble predictions and mean suitability maps further strengthens confidence in the major spatial patterns identified across scenarios.

## Climatic drivers of habitat suitability

The three species examined in this study are characteristic elements of temperate, sub-alpine, and alpine ecosystems of the Western Himalaya, where temperature regimes, seasonal moisture availability, and snow-related processes strongly influence plant distribution. *A. heterophyllum* typically occurs in moist sub-alpine meadows and forest openings, *L. polyphyllum* occupies cool temperate to sub-alpine habitats with relatively stable moisture conditions, whereas *P. kurroa* is largely restricted to alpine slopes and moist high-elevation grasslands^61,62^. Across species, precipitation seasonality (BIO15) and mean diurnal range (BIO2) emerged as the dominant environmental predictors, indicating that both moisture dynamics and thermal variability play central roles in determining habitat suitability.

The importance of precipitation seasonality highlights the ecological significance of monsoon-driven moisture regimes in Himalayan Mountain environments. Seasonal precipitation governs soil moisture availability, snow accumulation, snowmelt timing, and growing-season duration, all of which influence germination, growth, and reproductive success in alpine medicinal plants^48,63,64^. Snowmelt and seasonal moisture availability are particularly important in alpine ecosystems where short growing seasons strongly constrain plant establishment and persistence. Similar relationships have been reported for *P. kurroa* and other Himalayan alpine species, where moisture availability and snow-related processes are major determinants of species distribution and long-term persistence under climate change. Mean diurnal range reflects sensitivity to day–night temperature fluctuations, which affect physiological performance, carbon balance, and stress tolerance in high-elevation habitats. Previous studies have similarly identified moisture availability and thermal conditions as primary determinants of alpine plant distributions across the Himalaya.

Although the same climatic variables were important across species, their relative influences differed. *A. heterophyllum* was primarily associated with precipitation seasonality and mean diurnal range, indicating dependence on predictable seasonal moisture regimes and moderate thermal variability. Such conditions are characteristic of moist sub-alpine habitats where seasonal snowmelt and monsoon precipitation maintain favourable soil moisture conditions^65^. *L. polyphyllum* showed strong sensitivity to precipitation seasonality but exhibited suitability declines under increasing seasonality, suggesting a preference for relatively stable moisture conditions. This may explain its comparatively greater sensitivity to future changes in precipitation regimes projected under higher-emission scenarios^48^. *P. kurroa* was also strongly influenced by precipitation seasonality and mean diurnal range, reflecting adaptation to alpine environments characterized by pronounced seasonal climatic variation. Its strong dependence on alpine moisture and thermal regimes may contribute to the pronounced niche contraction projected under continued warming^66^. These differences demonstrate that species occupying similar elevational zones may nevertheless respond differently to changing climatic conditions.

## Species-specific responses to future climate change

Future projections indicate that all three species are likely to experience reductions and redistributions of climatically suitable habitat under future climate change, although the magnitude of change varies among taxa. Across scenarios, suitable habitats became increasingly concentrated within higher-elevation districts, while suitability declines in lower and mid-elevation areas.

Among the three species, *A. heterophyllum* exhibited the most moderate response, retaining relatively larger areas of suitable habitat under low- and intermediate-emission scenarios. This comparatively stable response may reflect the availability of climatically buffered habitats within topographically complex mountain landscapes. Similar persistence within mountain microrefugia has been reported for several Himalayan alpine and sub-alpine species occupying heterogeneous terrain^8,67^.

In contrast, *L. polyphyllum* showed greater redistribution and larger reductions in niche overlap under future scenarios, indicating higher climatic sensitivity. Despite currently occupying the broadest suitable area among the studied species, future projections suggest substantial contraction of suitable habitats under SSP245 and SSP585. *P. kurroa* retained suitable habitat under SSP126 but experienced marked contractions under stronger warming scenarios, particularly by 2070. These patterns suggest that even species currently occupying high-elevation environments may face increasing climatic constraints under continued warming.

The observed responses are consistent with previous studies demonstrating that many Himalayan medicinal plants possess relatively narrow niche breadths and restricted ecological amplitudes, increasing their vulnerability to climatic change and habitat restructuring^65^. Similar species distribution modelling studies from the Himalaya have likewise projected reductions in climatically suitable habitats and increasing concentration of suitable areas at higher elevations under future climate scenarios for several alpine medicinal and endemic plant species^48,68,69^. Our results broadly agree with these regional projections but further demonstrate that the magnitude of habitat contraction and redistribution differs considerably among the three focal medicinal plants despite their occurrence within similar high-elevation ecosystems. These species-specific differences highlight the importance of ecological specialization and climatic sensitivity in determining future responses to climate change and suggest that conservation planning should consider individual species rather than assuming uniform responses among Himalayan alpine medicinal plants.

## Distribution shifts and climatic refugia

Future suitability maps consistently indicate that climatically suitable habitats become increasingly concentrated within higher-elevation landscapes. This pattern is consistent with observations of upward elevational shifts reported for numerous Himalayan plant species under contemporary climate warming^58,70,71^. Previous Himalayan SDM studies have similarly reported progressive upslope redistribution and contraction of suitable habitats under future climate scenarios^48,72^. The present study extends these findings by combining ensemble suitability modelling with centroid displacement, niche overlap, and uncertainty analyses, thereby providing a more comprehensive assessment of climate-driven distributional change.

An important finding of this study is the distinction between local elevational shifts and regional centroid movement. While suitability maps suggest increasing concentration of suitable habitats at higher elevations, centroid analyses revealed a consistent south-westward displacement across species and scenarios. These patterns are not contradictory because they represent different aspects of distributional change. Elevational shifts describe local redistribution along mountain gradients, whereas centroid displacement reflects changes in the overall geographic centre of suitable habitat.

The south-westward movement of centroids likely results from asymmetric habitat loss across the species’ current ranges, causing the geometric centre of suitable habitat to shift even when remaining suitable areas occur at higher elevations^58,73^. The magnitude of centroid displacement varied among species, being smallest for *A. heterophyllum*, intermediate for *P. kurroa*, and greatest for *L. polyphyllum*. These differences indicate varying levels of sensitivity to climatic change and highlight how centroid metrics capture broader distributional restructuring beyond local elevational responses^58^. The larger centroid displacement observed for *L. polyphyllum* may be related to its broader current distribution across temperate and sub-alpine habitats, allowing greater spatial redistribution under changing climatic conditions. In contrast, *A. heterophyllum* exhibited smaller centroid movements because its current distribution is already concentrated within climatically suitable high-elevation environments, reducing the scope for large geographic displacement. *P. kurroa* occupied an intermediate position, showing moderate centroid movement despite habitat contraction, likely reflecting its strong association with alpine environments and limited opportunities for further upslope expansion.

## Niche contraction and ecological implications

A progressive decline in Schoener’s D was observed across all species and scenarios, indicating increasing divergence between present and future climatic niches. Declining niche overlap suggests that future suitable habitats may differ substantially from those currently occupied, reflecting changes not only in geographic distribution but also in the environmental conditions defining species persistence^74^. Comparable declines in climatic niche overlap and habitat suitability have also been reported for other Himalayan alpine plant species, supporting the view that niche contraction represents a widespread response of high-elevation flora to projected climate warming^48,65^.

This pattern is particularly important for alpine medicinal plants because climatic suitability alone may not guarantee long-term population persistence. Climate-driven changes in phenology, flowering periods, reproductive success, seedling establishment, and regeneration dynamics may further reduce viable populations even within areas projected to remain suitable. Recent studies have shown that warming can alter multiple ecological processes in alpine plant communities, including shifts in flowering phenology, changes in functional trait expression, reduced reproductive performance, and altered species interactions, thereby increasing vulnerability beyond that predicted by habitat suitability models alone^75^. Consequently, projected habitat persistence should not necessarily be interpreted as population stability, particularly for slow-growing Himalayan medicinal plants already facing pressures from habitat degradation and overharvesting^75^.

More broadly, most Himalayan climate-change studies have examined either species distributions or ecological responses independently. Integrating species distribution models with phenological observations, functional trait analyses, physiological performance measurements, reproductive ecology, regeneration studies, and demographic monitoring would provide a more mechanistic understanding of species vulnerability and adaptive capacity. Such approaches may improve future predictions by linking projected habitat changes with the biological processes that ultimately determine population persistence.

From a conservation perspective, the projected reductions in suitable habitat, declining niche overlap, and redistribution toward higher-elevation areas indicate that future conservation efforts should prioritize climatically stable mountain refugia and maintain ecological connectivity among suitable habitats. High-elevation districts such as Kullu, Kinnaur, and Lahaul & Spiti consistently emerged as areas of future climatic suitability and low model uncertainty, suggesting their importance for long-term conservation planning. However, future species persistence will depend not only on climatic suitability but also on habitat fragmentation, land-use change, grazing pressure, harvesting intensity, demographic processes, and dispersal limitations^65,76^. Integrating climate-informed species distribution models with ecological monitoring and habitat management will therefore be essential for supporting the long-term conservation of threatened Himalayan medicinal plants under ongoing climate change.

## Limitations

Future projections were based on a single General Circulation Model (HadGEM3-GC31-LL). Although this model has been widely applied in climate-impact assessments and provides high-resolution downscaled CMIP6 climate projections for the Himalayan region, the use of a single GCM does not capture the full range of uncertainty associated with differences among climate models. Consequently, the projected habitat suitability changes should be interpreted as representing one plausible future climate trajectory rather than the complete range of potential outcomes.

Future projections were generated using climatic variables while land-cover and soil layers were assumed to remain constant through time. Changes in land use, vegetation structure, infrastructure development, grazing pressure, and other environmental factors may alter habitat conditions and influence future species distributions.

Environmental predictors were analysed at a spatial resolution of approximately 1 km. Although suitable for regional-scale modelling, this resolution may not fully capture microclimatic heterogeneity, snow persistence, fine-scale topographic variation, or localized refugia that are particularly important in alpine environments.

The uncertainty analyses primarily reflect variation among modelling algorithms because future projections were generated using a single GCM. Consequently, they do not encompass uncertainty arising from alternative climate models. Future studies incorporating multi-model GCM ensembles, dynamic land-cover projections, high-resolution climate datasets, and microclimatic observations would provide a more comprehensive assessment of climate-related uncertainty and improve understanding of species responses to climate change in Himalayan mountain ecosystems. Nevertheless, the strong agreement among modelling algorithms within climatically suitable habitats suggests that the major spatial patterns identified in this study are robust, consistent with previous evaluations demonstrating the advantages of ensemble forecasting approaches in environmentally heterogeneous mountain systems^77^.

## Conclusion

This study used an ensemble species distribution modelling framework to assess the current and future climatic suitability of three threatened Himalayan medicinal plants, *A. heterophyllum*, *L. polyphyllum*, and *P. kurroa*, under multiple climate-change scenarios. The results indicate that precipitation seasonality and mean diurnal range are the primary environmental drivers shaping the distribution of all three species, highlighting the importance of seasonal moisture availability and thermal variability in Himalayan alpine and sub-alpine ecosystems. Future projections consistently indicate a redistribution of suitable habitats towards higher-elevation areas, accompanied by declining niche overlap with current climatic conditions and increasing habitat contraction under higher-emission scenarios. Although all species are projected to experience climate-driven range changes, the magnitude of response differs among species, reflecting differences in climatic sensitivity and ecological requirements.

The integration of ensemble suitability maps, centroid-shift analyses, niche-overlap metrics, and uncertainty assessments provided a comprehensive evaluation of future habitat dynamics. Areas identified as future high-suitability zones generally exhibited low model uncertainty, thereby, increasing confidence in the projected persistence of climatically suitable habitats within parts of the higher-elevation Western Himalaya. At the same time, increasing uncertainty under stronger emission scenarios highlights the growing unpredictability of species responses to future climate change.

Overall, the findings suggest that climate change is likely to substantially reshape both the geographic distribution and climatic niches of these medicinal plant species during the twenty-first century. Protecting climatically stable high-elevation habitats and maintaining landscape connectivity will be important for supporting long-term species persistence. The study also demonstrates the value of ensemble species distribution modelling for identifying potential climatic refugia and informing conservation planning for climate-sensitive Himalayan biodiversity.

## Supplementary Information

Supplementary Information 1.

Supplementary Information 2.

## Data Availability

The author confirms that all data generated or analyzed during this study are included in this published article. Furthermore, primary and secondary sources and data supporting the findings of this study were all publicly available at the time of submission.

## References

[1] Ioan, S., Roseo, F. & Brambilla, M. Mountain ecosystem services under a changing climate: A global perspective. *Ecosyst. Serv.* **73**, 101732 (2025).

[2] Malik, I. H. & Ford, J. D. Monitoring climate change vulnerability in the Himalayas. *Ambio* **54**, 1–19 (2025). 39215932 10.1007/s13280-024-02066-9 PMC11607287

[3] Matta, G., Kumar, A., Singh Tomar, D. & Kumar, R. Climate change in the Himalayan region: susceptible impacts on environment and human settlements. *Front. Environ. Sci.* **13**, (2025).

[4] Shaheen, H. et al. Distribution patterns of alpine flora for long-term monitoring of global change along a wide elevational gradient in the Western Himalayas. *Glob. Ecol. Conserv.* **48**, e02702 (2023).

[5] Bolch, T. et al. The state and fate of Himalayan glaciers. *Science* **1979**(336), 310–314 (2012). 10.1126/science.1215828 22517852

[6] Shrestha, U. B. et al. Climate change-induced distributional change of medicinal and aromatic plants in the Nepal Himalaya. *Ecol. Evol.* **12**, e9204 (2022). 35991283 10.1002/ece3.9204 PMC9379350

[7] Gaira, K. S., Rawal, R. S., Rawat, B. & Bhatt, I. D. Impact of climate change on the flowering of Rhododendron arboreum in central Himalaya, India. *Curr. Sci.* **106**, (2014).

[8] Gaira, K. S., Dhar, U. & Belwal, O. K. Potential of herbarium records to sequence phenological pattern: A case study of *Aconitum heterophyllum* in the Himalaya. *Biodivers. Conserv.* **20**, 2201–2210 (2011).

[9] Leng, R., Harrison, S. & Anderson, K. Himalayan alpine ecohydrology: An urgent scientific concern in a changing climate. *Ambio* **52**, 390–410 (2023). 36324019 10.1007/s13280-022-01792-2 PMC9755440

[10] Kattel, G. R. Climate warming in the Himalayas threatens biodiversity, ecosystem functioning and ecosystem services in the 21st century: is there a better solution? *Biodivers. Conserv. ***31** 2017–2044 Preprint at 10.1007/s10531-022-02417-6 (2022).

[11] Nomoto, H. A. & Alexander, J. M. Drivers of local extinction risk in alpine plants under warming climate. *Ecol. Lett.* **24**, 1157–1166 (2021). 33780124 10.1111/ele.13727 PMC7612402

[12] Myers‐Smith, I. H., Thomas, H. J. D. & Bjorkman, A. D. Plant traits inform predictions of tundra responses to global change. *New Phytol.* **221**, 1742–1748 (2019). 30444539 10.1111/nph.15592

[13] Maletha, A., Maikhuri, R. K. & Bargali, S. S. Population structure and regeneration pattern of Himalayan birch (*Betula utilis* D.Don) in the timberline zone of Nanda Devi Biosphere Reserve, Western Himalaya, India. *Geol. Ecol. Landscapes* **7**, 248–257 (2023).

[14] Eisenschmid, K., Jabbusch, S. & Koch, M. A. Evolutionary footprints of cold adaptation in arctic-alpine Cochlearia (Brassicaceae) – Evidence from freezing experiments and electrolyte leakage. *Perspect. Plant Ecol. Evol. Syst.* **59**, 125728 (2023).

[15] Liu, F.-L. et al. Spatiotemporal range dynamics and conservation optimization for endangered medicinal plants in the Himalaya. *Glob. Ecol. Conserv.* **57**, e03390 (2025).

[16] Mishra, A. P., Kumar, A. & Yadav, S. N. Ecology and conservation of threatened medicinal plants in the Trans-Himalayan region of Nanda Devi Biosphere Reserve, Western Himalaya. *Trees Forest. People* **14**, 100451 (2023).

[17] Wani, T. A., Kaloo, Z. A. & Dangroo, N. A. *Aconitum heterophyllum* Wall. ex Royle: A critically endangered medicinal herb with rich potential for use in medicine. *J. Integr. Med.* **20**, 104–113 (2022). 34996731 10.1016/j.joim.2021.12.004

[18] Dhyani, A., Kadaverugu, R., Nautiyal, B. P. & Nautiyal, M. C. Predicting the potential distribution of a critically endangered medicinal plant Lilium polyphyllum in Indian Western Himalayan Region. *Reg. Environ. Change* **21**, 30 (2021).

[19] McNichol, B. H. & Russo, S. E. Plant species’ capacity for range shifts at the habitat and geographic scales: a trade-off-based framework. *Plants* **12**, 1248 (2023). 36986935 10.3390/plants12061248 PMC10056461

[20] Fourcade, Y., Besnard, A. G. & Secondi, J. Paintings predict the distribution of species, or the challenge of selecting environmental predictors and evaluation statistics. *Glob. Ecol. Biogeogr.* **27**, 245–256 (2018).

[21] Hao, T., Elith, J., Guillera-Arroita, G. & Lahoz-Monfort, J. J. A review of evidence about use and performance of species distribution modelling ensembles like BIOMOD. *Diver. Distrib.* **25**, 839–852 (2019).

[22] Crimmins, S. M., Dobrowski, S. Z. & Mynsberge, A. R. Evaluating ensemble forecasts of plant species distributions under climate change. *Ecol. Modell.* **266**, 126–130 (2013).

[23] Abrahms, B. et al. Dynamic ensemble models to predict distributions and anthropogenic risk exposure for highly mobile species. *Divers. Distrib.* **25**, 1182–1193 (2019).

[24] Samant, S. S. et al. Medicinal plants in Himachal Pradesh, north western Himalaya, India. *Int. J. Biodivers. Sci. Manag.* **3**, 234–251 (2007).

[25] Kumar, P., Manikandan, R., Manikandan, R., Panwar, G. S. & Srivastava, S. Diversity of high altitude ethno-medicinal plants in Himachal Pradesh India. *Indian Forest.* **143**, 165–179 (2017).

[26] Haq, S. M. *et al.* Floristic composition, life history traits and phytogeographic distribution of forest vegetation in the Western Himalaya. *Front. Forest. Global Chang.* **6**, (2023).

[27] Haq, S. M., Waheed, M., Bussmann, R. W. & Kumar, M. Trouble in the rice field: Distribution ecology and indicator value of weed species in the rice fields of Himalayan region. *Acta Ecologica Sinica*10.1016/j.chnaes.2023.07.012 (2023) 10.1016/j.chnaes.2023.07.012.

[28] Chowdhary, H. J. & Wadhwa, B. M. *Flora of Himachal Pradesh*. (1984).

[29] Singh, P. & Kumar, N. Effect of orography on precipitation in the Western Himalayan region. *J. Hydrol. (Amst)* **199**, 183–206 (1997).

[30] Thakur, J., Dutt Sharma, D. & Chanjta, A. Assessing Climate Change Through Temperature and Rainfall Variability: A Case of Himachal Pradesh, India. In 59–76 10.1007/978-3-032-02118-2_3 (2025).

[31] Jreat, M. *Geography of Himachal Pradesh* (Indus Publishing Company, 2006).

[32] Das, J., Poonia, V., Jha, S. & Goyal, M. K. Understanding the climate change impact on crop yield over Eastern Himalayan region: Ascertaining GCM and scenario uncertainty. *Theor. Appl. Climatol.* **142**, 467–482 (2020).

[33] Panwar, S. Vulnerability of Himalayan springs to climate change and anthropogenic impact: A review. *J. Mt. Sci.* **17**, 117–132 (2020).

[34] Sharma, A., Batish, D. R. & Uniyal, S. K. Documentation and validation of climate change perception of an ethnic community of the Western Himalaya. *Environ. Monit. Assess.* **192**, 552 (2020). 32737629 10.1007/s10661-020-08512-x

[35] Barbet-Massin, M., Jiguet, F., Albert, C. H. & Thuiller, W. Selecting pseudo-absences for species distribution models: how, where and how many?. *Method. Ecol. Evol.* **3**, 327–338 (2012).

[36] Thuiller, W., Lafourcade, B., Engler, R. & Araújo, M. B. BIOMOD – a platform for ensemble forecasting of species distributions. *Ecography* **32**, 369–373 (2009).

[37] Thuiller, W., Georges, D. & Engler, R. Package “bio-mod 2” version 2.1.15. Preprint at https://biomodhub.github.io/biomod2/ (2012).

[38] Dormann, C. F. et al. Collinearity: a review of methods to deal with it and a simulation study evaluating their performance. *Ecography* **36**, 27–46 (2013).

[39] Swets, J. A. Measuring the accuracy of diagnostic systems. *Science* **1979**(240), 1285–1293 (1988). 10.1126/science.3287615 3287615

[40] Fielding, A. H. & Bell, J. F. A review of methods for the assessment of prediction errors in conservation presence/absence models. *Environ. Conserv.* **24**, 38–49 (1997).

[41] Guisan, A., Thuiller, W. & Zimmermann, N. E. *Habitat Suitability and Distribution Models* (Cambridge University Press, 2017). 10.1017/9781139028271.

[42] Elith, J., Ferrier, S., Huettmann, F. & Leathwick, J. The evaluation strip: A new and robust method for plotting predicted responses from species distribution models. *Ecol. Modell.* **186**, 280–289 (2005).

[43] Marmion, M., Parviainen, M., Luoto, M., Heikkinen, R. K. & Thuiller, W. Evaluation of consensus methods in predictive species distribution modelling. *Divers. Distrib.* **15**, 59–69 (2009).

[44] Araujo, M. & New, M. Ensemble forecasting of species distributions. *Trend. Ecol. Evol.* **22**, 42–47 (2007). 10.1016/j.tree.2006.09.010 17011070

[45] Baston, D. exactextractr: Fast extraction from raster datasets using polygons. Preprint at 10.32614/CRAN.package.exactextractr (2019).

[46] Fick, S. E. & Hijmans, R. J. WorldClim 2: new 1-km spatial resolution climate surfaces for global land areas. *Int. J. Climatol.* **37**, 4302–4315 (2017).

[47] Eyring, V. et al. Overview of the Coupled Model Intercomparison Project Phase 6 (CMIP6) experimental design and organization. *Geosci. Model Dev.* **9**, 1937–1958 (2016).

[48] Maharjan, S. K., Sterck, F. J., Raes, N. & Poorter, L. Temperature and soils predict the distribution of plant species along the Himalayan elevational gradient. *J. Trop. Ecol.* **38**, 58–70 (2022).

[49] Hijmans, R. J. *et al.* raster: Geographic data analysis and modeling. Preprint at (2025).

[50] Hijmans, R. J., Brown, A. & Barbosa, M. terra: Spatial data analysis. Preprint at (2026).

[51] Keitt, T., Bivand, R., Pebesma, E. & Rowlingson, B. rgdal: Bindings for the geospatial data abstraction library. (2010).

[52] Pebesma, E. & Bivand, R. sp: Classes and methods for spatial data. Preprint at 10.32614/CRAN.package.sp (2005).

[53] Wickham, H., François, R., Henry, L., Müller, K. & Vaughan, D. dplyr: A Grammar of Data Manipulation. Preprint at (2026).

[54] Wickham, H. *Ggplot2* (Springer International Publishing, 2016). 10.1007/978-3-319-24277-4.

[55] Greenwell, B. M. pdp: An R package for constructing partial dependence plots. *R. J.* **9**, 421 (2017).

[56] Phillips, S. J. & Dudík, M. Modeling of species distributions with Maxent: new extensions and a comprehensive evaluation. *Ecography* **31**, 161–175 (2008).

[57] Parmesan, C. & Yohe, G. A globally coherent fingerprint of climate change impacts across natural systems. *Nature* **421**, 37–42 (2003). 12511946 10.1038/nature01286

[58] Chen, I.-C., Hill, J. K., Ohlemüller, R., Roy, D. B. & Thomas, C. D. Rapid range shifts of species associated with high levels of climate warming. *Science* **1979**(333), 1024–1026 (2011). 10.1126/science.1206432 21852500

[59] Warren, D. L., Richard, E. G. & Michael, T. environmental niche equivalency versus conservatism: quantitative approaches to niche evolution. *Evolution (N. Y).* **62**, 1215–1215 (2008). 10.1111/j.1558-5646.2008.00482.x 18752605

[60] Elith, J. & Leathwick, J. R. Species distribution models: Ecological explanation and prediction across space and time. *Annu. Rev. Ecol. Evol. Syst.* **40**, 677–697 (2009).

[61] Samant, S. S. Forest types and diversity of fodder resource in Kumaun Himalaya proceeding of the seminar fodder problems faced by the Himalayan region in India. In *Proc. Seminar Fodder Problems Faces by the Himalayans Region in India* 111–123 (Lucknow, 1998).

[62] Kala, C. P. Indigenous uses, population density, and conservation of threatened medicinal plants in protected areas of the Indian Himalayas. *Conserv. Biol.* **19**, 368–378 (2005).

[63] Tomar, S., Thakur, S., Kanwal, K. S., Bhatt, I. D. & Puri, S. Population assessment and habitat suitability modelling of endangered medicinal plant, *Aconitum heterophyllum* Wall. ex Royle in the western Himalaya. *Sci. Rep.* **15**, 33794 (2025). 41028014 10.1038/s41598-025-03324-w PMC12484762

[64] Chandra, N., Singh, G., Lingwal, S., Bisht, M. P. S. & Tewari, L. M. Population Assessment and habitat distribution modelling of the threatened medicinal plant Picrorhiza Kurroa Royle Ex Benth. in the Kumaun Himalaya, India. *J. Threat. Taxa* **13**, 18868–18877 (2021).

[65] Manish, K. & Pandit, M. K. Identifying conservation priorities for plant species in the Himalaya in current and future climates: A case study from Sikkim Himalaya. *India. Biol. Conserv.* **233**, 176–184 (2019).

[66] Satti, Z., Naveed, M., Shafeeque, M. & Li, L. Investigating the impact of climate change on trend shifts of vegetation growth in Gilgit Baltistan. *Glob. Planet. Change* **232**, 104341 (2024).

[67] Salick, J., Fang, Z. & Byg, A. Eastern Himalayan alpine plant ecology, Tibetan ethnobotany, and climate change. *Glob. Environ. Change* **19**, 147–155 (2009).

[68] Ahmad, P. I. *et al.* Habitat suitability and niche modelling for conservation and restoration of aconitum heterophyllum wall. In Temperate Himalayan Forest Ecosystem. in *Ecosystem and Species Habitat Modeling for Conservation and Restoration* 227–247 10.1007/978-981-99-0131-9_12 (Springer Nature, 2023).

[69] Rawat, B. *et al.* A comprehensive review of Quercus semecarpifolia Sm.: An ecologically and commercially important Himalayan tree. *Front. Ecol. Evolut. ***10** Preprint at 10.3389/fevo.2022.961345 (2022).

[70] Telwala, Y., Brook, B. W., Manish, K. & Pandit, M. K. Climate-induced elevational range shifts and increase in plant species richness in a Himalayan biodiversity epicentre. *PLoS ONE*10.1371/journal.pone.0057103 (2013). 10.1371/journal.pone.0057103 PMC3577782 23437322

[71] Pecl, G. T. *et al.* Biodiversity redistribution under climate change: Impacts on ecosystems and human well-being. *Science (1979).* **355**, (2017). 10.1126/science.aai9214 28360268

[72] Ahmadi, M., Hemami, M., Kaboli, M. & Shabani, F. <scp>MaxEnt</scp> brings comparable results when the input data are being completed; Model parameterization of four species distribution models. *Ecol. Evol.* **13**, (2023). 10.1002/ece3.9827 PMC9937880 36820245

[73] Lenoir, J. & Svenning, J.-C. Climate-related range shifts – a global multidimensional synthesis and new research directions. *Ecography* **38**, 15–28 (2015).

[74] Wang, X. et al. Enhanced habitat loss of the Himalayan endemic flora driven by warming-forced upslope tree expansion. *Nat. Ecol. Evol.* **6**, 890–899 (2022). 35654898 10.1038/s41559-022-01774-3

[75] Sharma, M. K. & Chawla, A. Alpine plant species advance green-up but delay leaf senescence in response to experimental early snowmelt. *Plant Biol.* **28**, 304–313 (2026). 41100757 10.1111/plb.70121

[76] Schickhoff, U. et al. Do Himalayan treelines respond to recent climate change? An evaluation of sensitivity indicators. *Earth Syst. Dyn.* **6**, 245–265 (2015).

[77] Breiner, F. T., Guisan, A., Bergamini, A. & Nobis, M. P. Overcoming limitations of modelling rare species by using ensembles of small models. *Method. Ecol. Evol.* **6**, 1210–1218 (2015).

[78] Goraya, G. S. & Ved, D. K. CAMP’ Workshop (Conservation Assessment & Management Prioritization). In (Himachal Pradesh Forest Department and National Medicinal Plants Board, Government of India, Shimla, 2010).

[79] Dhyani, A., Kadaverugu, R., Nautiyal, B. P. & Nautiyal, M. C. Predicting the potential distribution of a critically endangered medicinal plant *Lilium polyphyllum* in Indian Western Himalayan region. *Reg. Environ. Change* **21**, 30 (2021).

[80] Wani, B. A. et al. Habitat suitability, range dynamics, and threat assessment of *Swertia petiolata* D. Don: A Himalayan endemic medicinally important plant under climate change. *Environ. Monit. Assess.* **195**, 214 (2022). 36538137 10.1007/s10661-022-10773-7

