# Supplementary material for: Projected climate change impacts on habitat suitability of threatened medicinal plants in Himachal Pradesh, Western Himalaya: an ensemble species distribution modelling approach
Source: Sci Rep. 2026 Aug 17;16:25724. doi: 10.1038/s41598-026-66433-0 (PMC13482990; doi:10.1038/s41598-026-66433-0)
Supplement: Supplementary file 1 [file 41598_2026_66433_MOESM1_ESM.docx]

**Table S1.** Sources of occurrence records and final sample sizes used for modelling.

| **Species** | **Field surveys** | **Herbarium records** | **Literature records** | **GBIF records** | **Initial total** | **Final cleaned records** |
| --- | --- | --- | --- | --- | --- | --- |
| *A. heterophyllum* | 51 | 05 | 00 | 26 | 82 | 73 |
| *L. polyphyllum* | 68 | 04 | 00 | 04 | 76 | 73 |
| *P. kurroa* | 82 | 03 | 00 | 10 | 95 | 77 |

**Table S2.** Environmental variables initially considered for species distribution modelling and their retention status following correlation and multicollinearity screening. Variables with pairwise Spearman correlation coefficients exceeding |r| = 0.70 were removed. Remaining predictors were evaluated using variance inflation factor (VIF) analysis, and all retained variables exhibited VIF values below 4.

| **Initial set of 24 variables** | **Variable removed/retained after correlation analysis** | **VIF** | **Ecological relevance** |
| --- | --- | --- | --- |
| Bio1 | Removed | VIF values >10 | - |
| Bio2 | Retained | 2.27 | Reflects daily temperature fluctuations affecting alpine plant physiology. |
| Bio3 | Retained | 3.58 | Represents thermal stability across seasons. |
| Bio4 | Removed | VIF values >10 | - |
| Bio5 | Removed | VIF values >10 | - |
| Bio6 | Removed | VIF values >10 | - |
| Bio7 | Removed | VIF values >10 | - |
| Bio8 | Removed | VIF values >10 | - |
| Bio9 | Removed | VIF values >10 | - |
| Bio10 | Removed | VIF values >10 | - |
| Bio12 | Removed | VIF values >10 | - |
| Bio13 | Removed | VIF values >10 | - |
| Bio14 | Retained | 3.33 | Indicates moisture limitation during stressful periods. |
| Bio15 | Retained | 2.72 | Captures variability in water availability. |
| Bio16 | Removed | VIF values >10 | - |
| Bio17 | Removed | VIF values >10 | - |
| Bio18 | Removed | VIF values >10 | - |
| Bio19 | Removed | VIF values >10 | - |
| Elevation | Removed | VIF values >10 | - |
| Slope | Retained | 1.70 | Influences drainage, snow retention and microhabitats. |
| Aspect | Retained | 1.00 | Controls solar radiation and snowmelt patterns. |
| Landcover | Retained | 1.83 | Represents vegetation and habitat suitability. |
| Soil | Retained | 1.36 | Influences rooting conditions and nutrient availability. |

**Figure S1.** Representative field photographs of the three focal species (a) *A. heterophyllum*, (b) *L. polyphyllum*, (c) *P. kurroa*.

a. ***A. heterophyllum***

**b. *L. polyphyllum***

**c. *P. kurroa***

**Figure S2**. Distribution of ROC and TSS values across six modelling algorithms for the three study species. Boxes indicate interquartile ranges, horizontal lines represent medians, whiskers represent 1.5 × IQR, and points indicate outliers.

**Figure S3**. Mean variable importance of environmental predictors for (a) *A. heterophyllum*, (b) *L. polyphyllum*, and (c) *P. kurroa*. Bio15 = precipitation seasonality; Bio14 = precipitation of driest month; Bio3 = isothermality; Bio2 = mean diurnal range.

a. ***A.*** ***heterophyllum***
b. ***L.*** ***polyphyllum***
c. ***P. kurroa***

**Figure S4**. Top three predictors (Bio15 = Precipitation seasonality; Bio2=Mean Diurnal Range; Bio14= Precipitation of Driest Month; Bio3= Isothermality) from the best-performing model of each algorithm based on ROC scores; (a) *A. heterophyllum;* (b) *L. polyphyllum;* (c) *P. kurroa*

a. ***A.*** ***heterophyllum***

Suitable habitat is concentrated in the high-elevation districts of Lahaul & Spiti, Chamba, Kullu and Kinnaur, whereas low-elevation districts contain negligible suitable area. Detailed district-wise suitability values are provided to complement the summary presented in the main text.

b. ***L.*** ***polyphyllum***

The species shows a broader distribution than the other taxa, with suitable habitat occurring across several mid- and high-elevation districts. Highest suitability is observed in Lahaul & Spiti and Kullu.

c. ***P. kurroa***

Suitable habitat is strongly concentrated in alpine districts, particularly Lahaul & Spiti, with limited occurrence in lower-elevation regions. This pattern reflects the species’ association with high-altitude environments

**Figure S5.** Graphical representation of current distribution of a. *A. heterophyllum, b. L. polyphyllum, c. P. kurroa* in each district of Himachal Pradesh under Unsuitable, Low, Medium and High suitable habitats. Blue region indicates no habitat suitability, yellow indicates poor habitat suitability, green indicates fair habitat suitability, purple indicates good habitat suitability. ArcMap 10.8 (<https://unikassel-its.maps.arcgis.com/home/>

**Table S3.** District-wise area (km²) of unsuitable, low, medium and high suitability habitat for the three species under current climatic conditions.

| **Species** | **Class** | **Total Current Area (km²)** |
| --- | --- | --- |
| *A. heterophyllum* | Unsuitable | 26809.52 |
|  | Low | 298.93 |
|  | Medium | 13553.51 |
|  | High | 13554.23 |
| *L. polyphyllum* | Unsuitable | 20342.06 |
|  | Low | 7476.01 |
|  | Medium | 13868.11 |
|  | High | 13871.06 |
| *P. Kurroa* | Unsuitable | 18631.97 |
|  | Low | 8874.55 |
|  | Medium | 13727.01 |
|  | High | 13697.12 |
