# Supplementary material for: Projected climate change impacts on habitat suitability of threatened medicinal plants in Himachal Pradesh, Western Himalaya: an ensemble species distribution modelling approach
Source: Sci Rep. 2026 Aug 17;16:25724. doi: 10.1038/s41598-026-66433-0 (PMC13482990; doi:10.1038/s41598-026-66433-0)
Supplement: Supplementary file 2 [file 41598_2026_66433_MOESM2_ESM.docx]

**Fig. S6(a)** *A. heterophyllum* mean suitability maps and Uncertainty maps under SSP126, 245 and 585 in 2050 and 2070

**2050**

**2070**

**SSP126**

**SSP245**

**SSP585**

**Fig. S6(b)** *L. polyphyllum* mean suitability maps and Uncertainty maps under SSP126, 245 and 585 in 2050 and 2070

**2050**

**2070**

**SSP126**

**SSP245**

**SSP585**

**2050**

**2070**

**SSP126**

**SSP245**

**SSP585**

**Fig. S6(c)** *P. Kurroa* mean suitability maps and Uncertainty maps under SSP126, 245 and 585 in 2050 and 2070

**Table. S4** Current and future distribution of A. heterophyllum, L. polyphyllum and P. kurroa in each district of Himachal Pradesh under the low, medium and high suitable habitats

| **Species** | **District** | **Suitability** | **Current** | **SSP126** | | **SSP245** | | **SSP585** | |
| --- | --- | --- | --- | --- | --- | --- | --- | --- | --- |
|  |  |  | **2025** | **2050** | **2070** | **2050** | **2070** | **2050** | **2070** |
| *L. polyphyllum* | Lahaul & Spiti | **Unsuitable** | 4509.54 | 6165.18 | 8527.61 | 1017.707 | 1208.89 | 1264.95 | 1068.02 |
|  |  | **Low** | 2402.93 | 4403.59 | 3557.66 | 8523.295 | 8781.31 | 7457.43 | 8833.06 |
|  |  | **Medium** | 3339.20 | 1382.10 | 719.44 | 695.720 | 769.03 | 865.34 | 669.85 |
|  |  | **High** | 3298.12 | 1553.15 | 699.31 | 3267.299 | 2744.79 | 3916.30 | 2933.09 |
|  |  | **Total Suitable Area** | 9040.25 | 7338.84 | 4976.41 | 12486.31 | 12295.13 | 12239.07 | 12436.00 |
|  | Shimla | **Unsuitable** | 1802.82 | 2563.67 | 2845.41 | 627.442 | 700.75 | 697.88 | 616.66 |
|  |  | **Low** | 789.36 | 2224.44 | 2058.41 | 3278.080 | 2790.79 | 2671.48 | 3009.28 |
|  |  | **Medium** | 878.38 | 81.22 | 56.06 | 338.517 | 436.26 | 449.92 | 388.11 |
|  |  | **High** | 1645.32 | 203.40 | 112.84 | 828.684 | 1144.92 | 1253.45 | 1058.67 |
|  |  | **Total Suitable Area** | 3313.06 | 2509.05 | 2227.31 | 4445.28 | 4371.97 | 4374.85 | 4456.06 |
|  | Chamba | **Unsuitable** | 2311.42 | 2506.17 | 3347.80 | 694.283 | 785.56 | 721.59 | 780.53 |
|  |  | **Low** | 643.69 | 3199.74 | 2606.08 | 2276.903 | 2918.00 | 2384.71 | 2281.22 |
|  |  | **Medium** | 1630.38 | 387.39 | 247.96 | 547.664 | 490.89 | 536.88 | 504.54 |
|  |  | **High** | 1793.48 | 278.86 | 170.34 | 2853.316 | 2177.72 | 2728.98 | 2805.88 |
|  |  | **Total Suitable Area** | 4067.55 | 3865.99 | 3024.37 | 5677.88 | 5586.61 | 5650.57 | 5591.64 |
|  | Hamirpur | **Unsuitable** | 111.43 | 0.00 | 0.00 | 51.748 | 32.34 | 24.44 | 7.19 |
|  |  | **Low** | 47.93 | 777.65 | 1026.33 | 350.016 | 91.28 | 98.46 | 32.34 |
|  |  | **Medium** | 960.55 | 191.90 | 61.81 | 45.998 | 51.03 | 37.37 | 11.50 |
|  |  | **High** | 2.49 | 148.06 | 29.47 | 669.847 | 942.96 | 957.33 | 1066.58 |
|  |  | **Total Suitable Area** | 1010.97 | 1117.61 | 1117.61 | 1065.86 | 1085.27 | 1093.17 | 1110.42 |
|  | Kinnaur | **Unsuitable** | 2327.61 | 3438.35 | 4179.35 | 436.981 | 644.69 | 674.16 | 551.26 |
|  |  | **Low** | 963.66 | 1859.33 | 1688.99 | 4109.638 | 4123.29 | 3599.35 | 3947.21 |
|  |  | **Medium** | 850.99 | 447.76 | 227.12 | 279.582 | 334.20 | 389.55 | 319.83 |
|  |  | **High** | 2152.68 | 502.38 | 152.37 | 1421.627 | 1145.64 | 1584.78 | 1429.53 |
|  |  | **Total Suitable Area** | 3967.33 | 2809.47 | 2068.47 | 5810.85 | 5603.14 | 5573.67 | 5696.57 |
|  | Una | **Unsuitable** | 755.74 | 129.37 | 310.49 | 40.248 | 94.15 | 80.50 | 112.12 |
|  |  | **Low** | 103.34 | 1264.95 | 1159.29 | 1247.697 | 278.14 | 245.08 | 306.17 |
|  |  | **Medium** | 603.85 | 62.53 | 12.94 | 25.874 | 69.72 | 81.93 | 99.90 |
|  |  | **High** | 41.09 | 28.03 | 2.16 | 171.055 | 1042.86 | 1077.36 | 966.68 |
|  |  | **Total Suitable Area** | 748.27 | 1355.50 | 1174.39 | 1444.63 | 1390.72 | 1404.38 | 1372.75 |
|  | Bilaspur | **Unsuitable** | 955.57 | 107.09 | 155.96 | 107.089 | 98.46 | 67.56 | 9.34 |
|  |  | **Low** | 148.78 | 875.40 | 901.99 | 560.601 | 431.95 | 210.58 | 56.06 |
|  |  | **Medium** | 36.73 | 108.53 | 65.40 | 89.840 | 89.12 | 70.43 | 19.41 |
|  |  | **High** | 0.00 | 51.03 | 18.69 | 384.515 | 522.51 | 793.47 | 1057.24 |
|  |  | **Total Suitable Area** | 185.51 | 1034.96 | 986.08 | 1034.96 | 1043.58 | 1074.49 | 1132.70 |
|  | Kangra | **Unsuitable** | 1739.32 | 541.91 | 893.37 | 382.359 | 411.83 | 479.39 | 340.67 |
|  |  | **Low** | 468.76 | 3648.94 | 3957.99 | 2750.540 | 1101.08 | 1584.78 | 916.37 |
|  |  | **Medium** | 2383.63 | 595.82 | 384.51 | 268.082 | 299.71 | 299.71 | 250.83 |
|  |  | **High** | 1021.56 | 825.09 | 375.89 | 2210.781 | 3799.15 | 3247.89 | 4103.89 |
|  |  | **Total Suitable Area** | 3873.95 | 5069.85 | 4718.39 | 5229.40 | 5199.94 | 5132.38 | 5271.09 |
|  | Kullu | **Unsuitable** | 319.98 | 1367.72 | 1947.01 | 534.727 | 693.56 | 641.82 | 664.82 |
|  |  | **Low** | 593.89 | 1832.73 | 2090.76 | 2227.312 | 2399.09 | 2121.66 | 2550.74 |
|  |  | **Medium** | 1187.15 | 871.09 | 717.28 | 339.236 | 403.20 | 413.98 | 389.55 |
|  |  | **High** | 3410.17 | 1442.47 | 758.97 | 2412.741 | 2018.16 | 2336.56 | 1908.92 |
|  |  | **Total Suitable Area** | 5191.20 | 4146.29 | 3567.00 | 4979.29 | 4820.45 | 4872.20 | 4849.20 |
|  | Mandi | **Unsuitable** | 2537.40 | 1062.99 | 1047.17 | 477.230 | 454.23 | 470.04 | 354.33 |
|  |  | **Low** | 742.05 | 2532.05 | 2557.92 | 1471.938 | 1124.80 | 1208.17 | 768.31 |
|  |  | **Medium** | 423.94 | 206.27 | 213.46 | 287.488 | 307.61 | 309.77 | 269.52 |
|  |  | **High** | 252.12 | 155.24 | 137.99 | 1719.896 | 2069.91 | 1968.57 | 2564.39 |
|  |  | **Total Suitable Area** | 1418.10 | 2893.56 | 2909.38 | 3479.32 | 3502.32 | 3486.51 | 3602.22 |
|  | Sirmaur | **Unsuitable** | 973.62 | 503.10 | 487.29 | 283.894 | 314.08 | 245.80 | 240.05 |
|  |  | **Low** | 305.66 | 2094.35 | 2174.13 | 1671.742 | 959.49 | 624.57 | 835.15 |
|  |  | **Medium** | 1385.73 | 81.93 | 44.56 | 180.399 | 222.80 | 208.43 | 196.21 |
|  |  | **High** | 90.27 | 37.37 | 10.78 | 580.725 | 1220.39 | 1637.96 | 1445.34 |
|  |  | **Total Suitable Area** | 1781.66 | 2213.66 | 2229.47 | 2432.87 | 2402.68 | 2470.96 | 2476.71 |
|  | Solan | **Unsuitable** | 1746.17 | 628.16 | 759.69 | 176.805 | 145.90 | 120.03 | 55.34 |
|  |  | **Low** | 150.65 | 1126.95 | 1014.83 | 1076.642 | 840.90 | 551.26 | 442.73 |
|  |  | **Medium** | 3.11 | 95.59 | 80.50 | 127.213 | 118.59 | 106.37 | 78.34 |
|  |  | **High** | 0.00 | 31.62 | 27.31 | 501.666 | 776.94 | 1104.67 | 1305.91 |
|  |  | **Total Suitable Area** | 153.76 | 1254.17 | 1122.64 | 1705.52 | 1736.43 | 1762.30 | 1826.98 |
| *A. heterophyllum* | Lahaul & Spiti | **Unsuitable** | 5671.96 | 4074.65 | 6729.37 | 5944.53 | 6944.53 | 6452.66 | 6535.32 |
|  |  | **Low** | 142.70 | 3499.74 | 3429.01 | 3416.07 | 3016.07 | 4486.97 | 3072.53 |
|  |  | **Medium** | 4937.42 | 1685.19 | 1161.45 | 1432.41 | 1422.41 | 1240.51 | 1415.16 |
|  |  | **High** | 2813.36 | 4243.53 | 2184.19 | 2711.01 | 2211.01 | 1323.88 | 2481.02 |
|  |  | **Total Suitable Area** | 7893.48 | 9428.46 | 6774.65 | 7559.49 | 6649.49 | 7051.36 | 6968.70 |
|  | Shimla | **Unsuitable** | 1285.04 | 771.09 | 1783.14 | 1001.89 | 1101.89 | 1106.11 | 725.91 |
|  |  | **Low** | 0.00 | 1047.77 | 1696.18 | 1726.36 | 1426.36 | 1898.86 | 1995.88 |
|  |  | **Medium** | 1448.79 | 498.73 | 566.35 | 799.93 | 699.93 | 826.53 | 836.59 |
|  |  | **High** | 2380.60 | 2755.96 | 1027.05 | 1544.53 | 1244.53 | 1241.23 | 1514.34 |
|  |  | **Total Suitable Area** | 3829.39 | 4302.45 | 3289.58 | 4070.83 | 3370.83 | 3966.61 | 4346.82 |
|  | Chamba | **Unsuitable** | 2176.30 | 735.16 | 2249.59 | 766.87 | 866.87 | 1863.64 | 588.63 |
|  |  | **Low** | 122.42 | 2589.23 | 3446.26 | 2407.71 | 2007.71 | 2910.10 | 3218.43 |
|  |  | **Medium** | 2632.46 | 1039.14 | 396.01 | 1092.45 | 1000.45 | 652.60 | 1035.67 |
|  |  | **High** | 1463.60 | 2007.86 | 280.30 | 2105.13 | 2005.13 | 945.83 | 1529.44 |
|  |  | **Total Suitable Area** | 4218.49 | 5636.24 | 4122.58 | 5605.29 | 5013.29 | 4508.53 | 5783.54 |
|  | Hamirpur | **Unsuitable** | 1124.41 | 740.19 | 1117.61 | 1103.95 | 1003.95 | 1116.17 | 1057.96 |
|  |  | **Low** | 0.00 | 378.00 | 0.00 | 13.66 | 10.66 | 1.44 | 59.65 |
|  |  | **Medium** | 0.00 | 0.00 | 0.00 | 0.00 | 0.00 | 0.00 | 0.00 |
|  |  | **High** | 0.00 | 0.00 | 0.00 | 0.00 | 0.00 | 0.00 | 0.00 |
|  |  | **Total Suitable Area** | 0.00 | 378.00 | 0.00 | 13.66 | 10.66 | 1.44 | 59.65 |
|  | Kinnaur | **Unsuitable** | 2215.29 | 1970.49 | 2967.59 | 2419.21 | 3419.21 | 2480.30 | 2136.03 |
|  |  | **Low** | 0.00 | 1334.50 | 1416.60 | 1300.88 | 1000.88 | 2215.09 | 1453.97 |
|  |  | **Medium** | 2272.21 | 648.21 | 568.51 | 615.22 | 315.22 | 683.50 | 622.41 |
|  |  | **High** | 1812.94 | 2293.16 | 1295.13 | 1912.51 | 1812.51 | 868.93 | 2035.41 |
|  |  | **Total Suitable Area** | 4085.15 | 4275.86 | 3280.24 | 3828.62 | 3128.62 | 3767.53 | 4111.79 |
|  | Una | **Unsuitable** | 1504.15 | 1451.64 | 1484.87 | 1484.16 | 2484.16 | 1478.41 | 1464.75 |
|  |  | **Low** | 0.00 | 34.49 | 0.00 | 0.72 | 0.52 | 6.47 | 14.37 |
|  |  | **Medium** | 0.00 | 0.00 | 0.00 | 0.00 | 0.00 | 0.00 | 3.59 |
|  |  | **High** | 0.00 | 0.00 | 0.00 | 0.00 | 0.00 | 0.00 | 2.16 |
|  |  | **Total Suitable Area** | 0.00 | 34.49 | 0.00 | 0.72 | 0.52 | 6.47 | 20.12 |
|  | Bilaspur | **Unsuitable** | 1156.38 | 804.87 | 1141.33 | 1121.20 | 1221.20 | 1103.23 | 1006.93 |
|  |  | **Low** | 0.00 | 327.70 | 0.72 | 17.25 | 14.25 | 31.62 | 97.75 |
|  |  | **Medium** | 0.00 | 4.31 | 0.00 | 1.44 | 1.41 | 5.03 | 24.44 |
|  |  | **High** | 0.00 | 4.31 | 0.00 | 2.16 | 1.12 | 2.16 | 12.94 |
|  |  | **Total Suitable Area** | 0.00 | 336.32 | 0.72 | 20.84 | 16.77 | 38.81 | 135.12 |
|  | Kangra | **Unsuitable** | 4422.00 | 3109.52 | 4345.38 | 3671.94 | 4671.94 | 3837.96 | 3555.51 |
|  |  | **Low** | 24.95 | 1070.76 | 585.76 | 804.97 | 604.97 | 835.87 | 856.71 |
|  |  | **Medium** | 351.67 | 190.44 | 208.43 | 329.89 | 229.89 | 282.46 | 323.42 |
|  |  | **High** | 860.07 | 1243.23 | 472.20 | 804.97 | 504.97 | 655.47 | 876.12 |
|  |  | **Total Suitable Area** | 1236.70 | 2504.43 | 1266.38 | 1939.82 | 1339.82 | 1773.80 | 2056.26 |
|  | Kullu | **Unsuitable** | 867.87 | 377.28 | 1174.39 | 495.20 | 699.20 | 733.81 | 294.67 |
|  |  | **Low** | 8.58 | 748.10 | 1307.35 | 1500.69 | 1400.69 | 2149.69 | 1754.39 |
|  |  | **Medium** | 948.18 | 405.31 | 534.73 | 918.52 | 718.52 | 928.59 | 950.87 |
|  |  | **High** | 3702.29 | 3981.94 | 2497.55 | 2599.61 | 2199.61 | 1701.93 | 2514.08 |
|  |  | **Total Suitable Area** | 4659.05 | 5135.35 | 4339.63 | 5018.82 | 4318.82 | 4780.20 | 5219.34 |
|  | Mandi | **Unsuitable** | 2337.71 | 570.59 | 2308.53 | 1182.29 | 1582.29 | 1406.53 | 774.78 |
|  |  | **Low** | 17.15 | 1213.05 | 985.36 | 1557.47 | 1357.47 | 1180.14 | 1714.86 |
|  |  | **Medium** | 929.47 | 470.70 | 258.74 | 436.26 | 400.26 | 511.01 | 614.51 |
|  |  | **High** | 680.73 | 1701.72 | 403.92 | 780.53 | 711.53 | 858.87 | 852.40 |
|  |  | **Total Suitable Area** | 1627.35 | 3385.48 | 1648.02 | 2774.26 | 2469.26 | 2550.02 | 3181.77 |
|  | Sirmaur | **Unsuitable** | 2650.40 | 2250.76 | 2560.80 | 2318.59 | 2518.59 | 2216.53 | 1996.60 |
|  |  | **Low** | 0.00 | 263.02 | 140.15 | 212.74 | 199.74 | 264.49 | 369.42 |
|  |  | **Medium** | 79.54 | 48.15 | 12.22 | 79.06 | 69.06 | 115.71 | 189.74 |
|  |  | **High** | 31.97 | 155.22 | 3.59 | 106.37 | 99.37 | 120.03 | 160.99 |
|  |  | **Total Suitable Area** | 111.51 | 466.39 | 155.96 | 398.17 | 368.17 | 500.23 | 720.16 |
|  | Solan | **Unsuitable** | 1877.66 | 1080.82 | 1854.30 | 1691.15 | 1891.15 | 1571.12 | 1347.60 |
|  |  | **Low** | 0.00 | 633.83 | 28.03 | 120.03 | 98.03 | 165.31 | 227.12 |
|  |  | **Medium** | 26.51 | 87.67 | 0.00 | 36.65 | 16.65 | 73.31 | 158.12 |
|  |  | **High** | 0.00 | 80.49 | 0.00 | 34.50 | 24.50 | 72.59 | 149.49 |
|  |  | **Total Suitable Area** | 26.51 | 801.99 | 28.03 | 191.18 | 139.18 | 311.21 | 534.73 |
| *P. Kurroa* | Lahaul & Spiti | **Unsuitable** | 1544.47 | 5471.61 | 6974.45 | 5942.37 | 7264.10 | 6364.98 | 6278.01 |
|  |  | **Low** | 3029.81 | 4630.71 | 3972.36 | 3216.99 | 3913.43 | 4308.72 | 3316.89 |
|  |  | **Medium** | 4014.02 | 1639.40 | 1308.07 | 1400.07 | 1186.61 | 1476.25 | 1319.57 |
|  |  | **High** | 4961.49 | 1762.30 | 1249.13 | 2944.59 | 1139.89 | 1354.07 | 2589.55 |
|  |  | **Total Suitable Area** | 12005.32 | 8032.41 | 6529.57 | 7561.65 | 6239.92 | 7139.04 | 7226.01 |
|  | Shimla | **Unsuitable** | 285.11 | 1230.45 | 1840.64 | 830.84 | 1078.08 | 927.15 | 710.09 |
|  |  | **Low** | 706.56 | 2479.58 | 2519.83 | 2228.03 | 2023.20 | 2036.85 | 2511.92 |
|  |  | **Medium** | 2659.41 | 712.97 | 403.20 | 935.05 | 812.15 | 835.15 | 896.24 |
|  |  | **High** | 1464.79 | 649.72 | 309.05 | 1078.80 | 1159.29 | 1273.57 | 954.46 |
|  |  | **Total Suitable Area** | 4830.76 | 3842.27 | 3232.08 | 4241.88 | 3994.64 | 4145.57 | 4362.63 |
|  | Chamba | **Unsuitable** | 1688.90 | 2044.04 | 2589.55 | 1437.44 | 2320.03 | 1481.28 | 1814.05 |
|  |  | **Low** | 1452.34 | 2670.76 | 2701.67 | 2296.31 | 2991.31 | 3201.18 | 2708.14 |
|  |  | **Medium** | 1697.61 | 1026.33 | 718.00 | 931.46 | 685.66 | 981.77 | 869.65 |
|  |  | **High** | 1540.12 | 631.04 | 362.95 | 1706.96 | 375.17 | 707.94 | 980.33 |
|  |  | **Total Suitable Area** | 4690.07 | 4328.13 | 3782.62 | 4934.73 | 4052.14 | 4890.89 | 4558.12 |
|  | Hamirpur | **Unsuitable** | 1122.41 | 1117.61 | 1117.61 | 1117.61 | 1117.61 | 1117.61 | 1093.17 |
|  |  | **Low** | 0.00 | 0.00 | 0.00 | 0.00 | 0.00 | 0.00 | 24.44 |
|  |  | **Medium** | 0.00 | 0.00 | 0.00 | 0.00 | 0.00 | 0.00 | 0.00 |
|  |  | **High** | 0.00 | 0.00 | 0.00 | 0.00 | 0.00 | 0.00 | 0.00 |
|  |  | **Total Suitable Area** | 0.00 | 0.00 | 0.00 | 0.00 | 0.00 | 0.00 | 24.44 |
|  | Kinnaur | **Unsuitable** | 1074.47 | 2209.34 | 2736.17 | 2073.51 | 2603.92 | 2346.62 | 1893.83 |
|  |  | **Low** | 1358.96 | 2137.47 | 2409.15 | 2054.10 | 2281.93 | 2087.16 | 2406.27 |
|  |  | **Medium** | 2394.84 | 794.90 | 647.57 | 724.47 | 636.07 | 687.81 | 829.40 |
|  |  | **High** | 1466.66 | 1106.11 | 454.95 | 1395.75 | 725.91 | 1126.23 | 1118.33 |
|  |  | **Total Suitable Area** | 5220.46 | 4038.48 | 3511.66 | 4174.32 | 3643.91 | 3901.21 | 4354.00 |
|  | Una | **Unsuitable** | 1504.01 | 1484.87 | 1484.87 | 1484.87 | 1484.87 | 1482.72 | 1469.06 |
|  |  | **Low** | 0.00 | 0.00 | 0.00 | 0.00 | 0.00 | 2.16 | 15.81 |
|  |  | **Medium** | 0.00 | 0.00 | 0.00 | 0.00 | 0.00 | 0.00 | 0.00 |
|  |  | **High** | 0.00 | 0.00 | 0.00 | 0.00 | 0.00 | 0.00 | 0.00 |
|  |  | **Total Suitable Area** | 0.00 | 0.00 | 0.00 | 0.00 | 0.00 | 2.16 | 15.81 |
|  | Bilaspur | **Unsuitable** | 1141.08 | 1141.33 | 1142.05 | 1129.83 | 1136.30 | 1117.61 | 1055.08 |
|  |  | **Low** | 0.00 | 0.72 | 0.00 | 12.22 | 5.75 | 24.44 | 86.97 |
|  |  | **Medium** | 0.00 | 0.00 | 0.00 | 0.00 | 0.00 | 0.00 | 0.00 |
|  |  | **High** | 0.00 | 0.00 | 0.00 | 0.00 | 0.00 | 0.00 | 0.00 |
|  |  | **Total Suitable Area** | 0.00 | 0.72 | 0.00 | 12.22 | 5.75 | 24.44 | 86.97 |
|  | Kangra | **Unsuitable** | 4254.31 | 4287.16 | 4375.56 | 3949.36 | 4026.99 | 3858.80 | 3843.71 |
|  |  | **Low** | 282.00 | 423.33 | 471.48 | 687.10 | 836.59 | 986.80 | 974.58 |
|  |  | **Medium** | 351.10 | 301.14 | 307.61 | 356.48 | 390.26 | 449.92 | 360.08 |
|  |  | **High** | 725.86 | 600.13 | 457.11 | 618.82 | 357.92 | 316.24 | 433.39 |
|  |  | **Total Suitable Area** | 1358.96 | 1324.60 | 1236.20 | 1662.40 | 1584.78 | 1752.96 | 1768.05 |
|  | Kullu | **Unsuitable** | 64.74 | 733.09 | 1216.07 | 488.73 | 838.03 | 472.20 | 396.73 |
|  |  | **Low** | 670.46 | 1347.60 | 1453.25 | 1524.40 | 1777.39 | 1707.68 | 1796.80 |
|  |  | **Medium** | 1566.26 | 716.56 | 787.00 | 1119.05 | 986.80 | 1003.33 | 1254.88 |
|  |  | **High** | 3209.72 | 2716.76 | 2057.69 | 2381.84 | 1911.79 | 2330.81 | 2065.60 |
|  |  | **Total Suitable Area** | 5446.44 | 4780.92 | 4297.94 | 5025.29 | 4675.99 | 5041.82 | 5117.28 |
|  | Mandi | **Unsuitable** | 1965.30 | 1824.11 | 2504.74 | 1535.18 | 1483.44 | 1224.70 | 1112.58 |
|  |  | **Low** | 815.50 | 1671.02 | 1114.73 | 1714.15 | 1554.59 | 1805.42 | 2330.09 |
|  |  | **Medium** | 835.42 | 224.96 | 150.93 | 321.99 | 304.74 | 324.86 | 252.27 |
|  |  | **High** | 339.27 | 236.46 | 186.15 | 385.23 | 613.79 | 601.57 | 261.61 |
|  |  | **Total Suitable Area** | 1990.20 | 2132.44 | 1451.81 | 2421.37 | 2473.11 | 2731.85 | 2843.97 |
|  | Sirmaur | **Unsuitable** | 2225.51 | 2598.89 | 2646.33 | 2358.84 | 2419.21 | 2329.37 | 2138.91 |
|  |  | **Low** | 374.76 | 115.00 | 68.28 | 342.83 | 260.90 | 370.14 | 577.13 |
|  |  | **Medium** | 154.39 | 2.87 | 2.16 | 10.06 | 21.56 | 9.34 | 0.72 |
|  |  | **High** | 0.62 | 0.00 | 0.00 | 5.03 | 15.09 | 7.91 | 0.00 |
|  |  | **Total Suitable Area** | 529.77 | 117.87 | 70.43 | 357.92 | 297.55 | 387.39 | 577.85 |
|  | Solan | **Unsuitable** | 1700.10 | 1806.86 | 1865.80 | 1668.15 | 1756.55 | 1655.93 | 1432.41 |
|  |  | **Low** | 169.95 | 75.47 | 16.53 | 214.18 | 125.78 | 226.40 | 449.92 |
|  |  | **Medium** | 29.88 | 0.00 | 0.00 | 0.00 | 0.00 | 0.00 | 0.00 |
|  |  | **High** | 0.00 | 0.00 | 0.00 | 0.00 | 0.00 | 0.00 | 0.00 |
|  |  | **Total Suitable Area** | 199.83 | 75.47 | 16.53 | 214.18 | 125.78 | 226.40 | 449.92 |

**Table S5.** Centroid shift and change in Bearing degrees for all the three species calculated on the basis of change in latitudes and longitudes and Schoener D index.

| **Species** | **SSP** | **Year** | **Area (km^2^)** | **Centroid**  **longitude** | **Centroid latitude** | **Shift**  **(km)** | **Bearing**  **degrees** | **Schoener D** | **Current Area (km^2^)** | **Current Centroid longitude** | **Current**  **Centroid latitude** |
| --- | --- | --- | --- | --- | --- | --- | --- | --- | --- | --- | --- |
| *A. heterophyllum* | SSP126 | 2050 | 20952929.64 | 77.28779309 | 31.93334907 | 11.57 | -125.001 | 0.55749671 | 15444375 | 77.38809 | 31.99328 |
|  |  | 2070 | 88869.07145 | 77.29174266 | 31.97248714 | 9.39 | -104.185 | 0.543524 | 15444375 | 77.38809 | 31.99328 |
|  | SSP245 | 2050 | 99868.79242 | 77.26647286 | 31.97902267 | 11.60 | -97.801 | 0.508476 | 15444375 | 77.38809 | 31.99328 |
|  |  | 2070 | 99868.79242 | 77.26647286 | 31.97902267 | 11.60 | -97.801 | 0.508476 | 15444375 | 77.38809 | 31.99328 |
|  | SSP585 | 2050 | 92956.17777 | 77.27576868 | 31.95129107 | 11.59 | -113.651 | 0.496031 | 15444375 | 77.38809 | 31.99328 |
|  |  | 2070 | 101202.382 | 77.26841481 | 31.95364413 | 12.13 | -111.202 | 0.489072 | 15444375 | 77.38809 | 31.99328 |
| *L. polyphyllum* | ssp126 | 2050 | 97101.73 | 77.17866 | 31.92989 | 16.69629 | -139.123 | 0.586554 | 20675880 | 77.2942 | 32.04379 |
|  |  | 2070 | 88819.31 | 77.16745 | 31.90621 | 19.39827 | -141.824 | 0.56203 | 20675880 | 77.2942 | 32.04379 |
|  | ssp245 | 2050 | 131555.3 | 77.216 | 31.93714 | 13.94593 | -147.978 | 0.560729 | 20675880 | 77.2942 | 32.04379 |
|  |  | 2070 | 134642.8 | 77.19493 | 31.91498 | 17.0893 | -146.673 | 0.535048 | 20675880 | 77.2942 | 32.04379 |
|  | ssp585 | 2050 | 137794.3 | 77.20341 | 31.91644 | 16.5239 | -148.69 | 0.543831 | 20675880 | 77.2942 | 32.04379 |
|  |  | 2070 | 137229.1 | 77.18691 | 31.91279 | 17.71638 | -145.053 | 0.528566 | 20675880 | 77.2942 | 32.04379 |
| *P. kurroa* | ssp126 | 2050 | 92339.54 | 77.30045 | 31.97449 | 13.40195 | -129.925 | 0.503475 | 17218614 | 77.40919 | 32.0521 |
|  |  | 2070 | 85150.19 | 77.28722 | 31.97366 | 14.43746 | -127.011 | 0.502977 | 17218614 | 77.40919 | 32.0521 |
|  | ssp245 | 2050 | 96971.39 | 77.28252 | 31.98202 | 14.26847 | -122.963 | 0.475527 | 17218614 | 77.40919 | 32.0521 |
|  |  | 2070 | 89270.77 | 77.28826 | 31.94668 | 16.34651 | -135.618 | 0.478529 | 17218614 | 77.40919 | 32.0521 |
|  | ssp585 | 2050 | 93860.12 | 77.28419 | 31.95751 | 15.79577 | -131.574 | 0.472394 | 17218614 | 77.40919 | 32.0521 |
|  |  | 2070 | 95675.62 | 77.2915 | 31.95925 | 15.15426 | -132.764 | 0.467589 | 17218614 | 77.40919 | 32.0521 |
